# Author Correction: Factor XII signaling via uPAR-integrin β1 axis promotes tubular senescence in diabetic kidney disease

**DOI:** 10.1038/s41467-026-68442-z

**Published:** 2026-01-14

**Authors:** Ahmed Elwakiel, Dheerendra Gupta, Rajiv Rana, Jayakumar Manoharan, Moh’d Mohanad Al-Dabet, Saira Ambreen, Sameen Fatima, Silke Zimmermann, Akash Mathew, Zhiyang Li, Kunal Singh, Anubhuti Gupta, Surinder Pal, Alba Sulaj, Stefan Kopf, Constantin Schwab, Ronny Baber, Robert Geffers, Tom Götze, Bekas Alo, Christina Lamers, Paul Kluge, Georg Kuenze, Shrey Kohli, Thomas Renné, Khurrum Shahzad, Berend Isermann

**Affiliations:** 1 Institute of Laboratory Medicine, Clinical Chemistry and Molecular Diagnostics, University of Leipzig Medical Center, Leipzig, Germany; 2 Internal Medicine I and Clinical Chemistry, German Diabetes Center (DZD), University of Heidelberg, Heidelberg, Germany; 3 Institute of pathology, University of Heidelberg, Heidelberg, Germany; 4 Leipzig Medical Biobank, Leipzig University, Leipzig, Germany; 5 Genome Analytics, Helmholtz Centre for Infection Research, Braunschweig, Germany; 6 Institute for Drug Discovery, Faculty of Medicine, Leipzig University, Leipzig, Germany; 7 Center for Scalable Data Analytics and Artificial Intelligence, Leipzig University, Leipzig, Germany; 8 Institute of Clinical Chemistry and Laboratory Medicine, University Medical Center Hamburg-Eppendorf, Hamburg, Germany; 9 Center for Thrombosis and Hemostasis (CTH), Johannes Gutenberg University Medical Center, Mainz, Germany; 10 Irish Centre for Vascular Biology, School of Pharmacy and Biomolecular Sciences, Royal College of Surgeons in Ireland, Dublin, Ireland; 11 National Centre of Excellence in Molecular Biology, University of the Punjab, 87-West Canal Bank Road, Lahore, Pakistan; 12 Present Address: Department of Medical Laboratory Sciences, School of Science, University of Jordan, Amman, Jordan

**Keywords:** Chronic kidney disease, Molecular biology, Risk factors, Experimental models of disease

Correction to: *Nature Communications* 10.1038/s41467-024-52214-8, published online 11 September 2024

In the version of this article initially published, the Methods research ethics statement was incomplete and has now been amended to read “The research conducted in this study complies with the ethical regulations of the University of Leipzig (Ethic vote no: 263-2009-14122009 and 201/17-ek), the University of Heidelberg (Ethic vote no: S-383/2016), the confirmation of the related notification by the local animal care and use committee (T01_20; Landesdirektion, Leipzig, Germany), the local animal care and use committee (Landesverwaltungsamt, Halle, Germany), and the local animal care and use committee (Heidelberg, Germany)” in the HTML and PDF versions of the article.

