# Supplementary material for: Factor XII signaling via uPAR-integrin β1 axis promotes tubular senescence in diabetic kidney disease
Source: Nat Commun. 2024 Sep 11;15:7963. doi: 10.1038/s41467-024-52214-8 (PMC11390906; doi:10.1038/s41467-024-52214-8)
Supplement: Supplementary file 1 — Supplementary Information [file 41467_2024_52214_MOESM1_ESM.pdf]

Factor XII signaling via uPAR-integrin β1 axis promotes tubular senescence in diabetic kidney disease

Ahmed Elwakiel[1], Dheerendra Gupta[1], Rajiv Rana[1], Jayakumar Manoharan[1], Moh'd Mohanad Al-Dabet[1,12], Saira Ambreen[1], Sameen Fatima[1], Silke Zimmermann[1], Akash Mathew[1], Zhiyang Li[1], Kunal Singh[1], Anubhuti Gupta[1], Surinder Pal[1], Alba Sulaj[2], Stefan Kopf[2], Constantin Schwab[3], Ronny Baber[1,4], Robert Geffers[5], Tom Götze[6], Bekas Alo[6], Christina Lamers[6], Paul Kluge[6], Georg Kuenze[6,7], Shrey Kohli[1], Thomas Renné [8,9,10], Khurrum Shahzad[1,11], Berend Isermann[1*]

[1] Institute of Laboratory Medicine, Clinical Chemistry and Molecular Diagnostics, University of Leipzig Medical Center, Leipzig, Germany.

[2] Internal Medicine I and Clinical Chemistry, German Diabetes Center (DZD), University of Heidelberg, Heidelberg, Germany.

[3] Institute of pathology, University of Heidelberg, Heidelberg, Germany.

[4] Leipzig Medical Biobank, Leipzig University, Leipzig, Germany.

[5] Genome Analytics, Helmholtz Centre for Infection Research, Braunschweig, Germany.

[6] Institute for Drug Discovery, Faculty of Medicine, Leipzig University, Leipzig, Germany.

[7] Center for Scalable Data Analytics and Artificial Intelligence, Leipzig University, Leipzig, Germany.

[8] Institute of Clinical Chemistry and Laboratory Medicine, University Medical Center Hamburg-Eppendorf, Hamburg, Germany.

[9] Center for Thrombosis and Hemostasis (CTH), Johannes Gutenberg University Medical Center, Mainz, Germany.

[10] Irish Centre for Vascular Biology, School of Pharmacy and Biomolecular Sciences, Royal College of Surgeons in Ireland, Dublin, Ireland.

[11] National Centre of Excellence in Molecular Biology, University of the Punjab, 87-West Canal Bank Road, Lahore, Pakistan.

[12] Department of Medical Laboratory Sciences, School of Science, University of Jordan, Amman, Jordan (current affiliation).

*Corresponding author:

Berend Isermann, MD

Institute of Laboratory Medicine, Clinical Chemistry and Molecular Diagnostics

University hospital Leipzig

Paul-List-Straße 13/15, 04103 Leipzig, Germany

Phone: +49 – (0)341-97-22200

Fax: +49 – (0)341-97-22379

E-mail: berend.isermann@medizin.uni-leipzig.de

Inventory of supporting information

Supplementary methods

Supplementary tables:

Table S1

Table S2

Table S3

Table S4

Table S5

Table S6

Supplementary references

Supplementary Figures:

Fig. S1

Fig. S2

Fig. S3

Fig. S4

Fig. S5

Fig. S6

Fig. S7

Fig. S8

Fig. S9

Fig. S10

Fig. S11

Fig. S12

Fig. S13

Fig. S14

Fig. S15

Fig. S16

Fig. S17

Fig. S18

Fig. S19

Fig. S20

Fig. S21

Fig. S22

Supplementary methods:

Reagents

Antibodies: mouse anti-human FXII (clone C-8; sc-376770), mouse anti-Integrin β1 (clone 12G10; sc-59827), and mouse anti-Integrin β1 (clone P5D2; sc-59827), mouse anti-Kininogen HC (clone H-5; sc-166631), rat anti-Integrin a6 (clone GOH3; sc-19622), rabbit anti- Integrin a5 (clone H-104; sc-10729) (Santa Cruz Biotechnology, Germany); rabbit anti-human uPAR (clone D4Q5S; 12863), rabbit anti-human p21 (clone 12D1; 2947), rabbit anti-γ-H2A.X (clone 20E3; 9718), rabbit anti-Ki-67 (clone D3B5; 9129), rabbit anti-Lamin B1 (clone E6M5T; 17416), rabbit anti-Bcl-2 (clone D17C4; 3498), rabbit anti-Bcl-xL (clone 54H6; 2764), rabbit anti-phospho-FAK (Tyr397; 3283), rabbit anti-FAK (3285), rabbit anti-phospho-Src (Tyr416; clone D49G4; 6943), rabbit anti-Src (2108); rabbit anti-Rac1/Cdc42 (4651); rabbit anti-α-Tubulin (clone 11H10; 2125), rabbit anti-β-Actin (clone 13E5; 4970); goat anti-rabbit IgG HRP-linked (7074), horse anti-mouse IgG HRP-linked (7076), goat anti-rat IgG HRP-linked (7077), mouse anti-rabbit IgG (conformation specific) HRP-linked (clone L27A9; 3678), rabbit anti-mouse IgG (light-chain specific) HRP-linked (clone D3V2A; 58802) (Cell Signaling Technology, Germany); rabbit anti-NOX1 (PA1739) (BOSTER, USA); rabbit anti-KIM-1 (ab47635) and rabbit anti-mouse p21 (clone EPR18021; ab188224), rat anti-F4/80 (clone BM8; ab16911) (Abcam, USA); rabbit anti-uPAR (orb13650) and rabbit anti-FXII (orb48369) (Biorbyt, UK); goat anti-mouse uPAR (AF534), mouse anti- 8-O-dG (clone 15A3; 4354-MC-050), VisUCyte HRP polymer mouse/rabbit IgG antibody (VC002-025) (R&D systems, USA); rabbit anti-WT-1 (clone CEH-23; MAB20854; Abnova, Taiwan); mouse anti- Integrin β3 (clone AP-5; EBW107; Kerafast, USA); mouse anti-Integrin β3 (clone B3A; MAB2023Z; Sigma-Aldrich, Germany); rat anti-mouse Integrin β1 (clone 9EG7; 553715; BD Biosciences, Germany); rat anti-mouse uPAR (clone 109801; MA5-23853), DyLight™ 649 donkey anti-rabbit IgG (4064; Biolegend, USA); Alexa Fluor™ 568 donkey anti-rabbit IgG (A10042), Alexa Fluor™ Plus 647 donkey anti-rabbit IgG (A32795), Alexa Fluor™ 488 donkey anti-mouse IgG (A-21202), Alexa Fluor™ 568 anti-mouse IgG (A10037), Alexa Fluor™ 488 Phalloidin (A12379) (Thermo Fischer Scientific, Germany).

Other reagents: human FXII CRISPR/Cas9 KO plasmid (sc-409611; Santa Cruz Biotechnology, Germany); Dulbecco's Modified Eagle's Medium (DMEM), Ham's F-12 nutrient mixture, trypsin-EDTA, penicillin/streptomycin solution, fetal bovine serum (FBS), ITS supplement, D(+)-Glucose, type I collagenase, RNAlater™, Hank's Balanced Salt Solution (HBSS) and HEPES buffer (PAA Laboratories, Austria); donkey serum, horse serum, antibiotic/antimycotic solution, APO transferrin and hydrocortisone solution, Duolink® in situ PLA® probes (anti-rabbit plus, anti-goat plus, anti-goat minus, anti-mouse minus), Duolink® in situ probemaker plus, Duolink® in situ mounting medium with DAPI and Duolink® in situ detection reagents red (Sigma-Aldrich, Germany); recombinant human epidermal growth factor (R&D systems, USA); IFN-γ (Cell Sciences, Germany); BCA reagent (Perbio Science, Germany); Vectashield mounting medium with DAPI and citrate-based antigen unmasking solution (Vector Laboratories, USA); PVDF membranes and immobilon TM western chemiluminescent HRP substrate (Millipore, USA); ammonium persulphate (Merck, Germany); streptozotocin (Enzo Life Sciences, Germany); insulin glargine (Lantus®, Sanofi, France); accu-chek test strips, accu-check glucometer and protease inhibitor cocktail (Roche Diagnostics, Germany); albumin fraction V, hematoxylin Gill II, acrylamide, agarose, periodic acid-Schiff (PAS) reagent, aqueous Eosin, Histokitt synthetic mounting medium, zinc chloride and dimethylsulfoxide (Carl ROTH, Germany); Trizol Reagent and phosphate buffer saline (Life Technologies, Germany); mouse FXII vivo morpholino and FXII mismatch vivo morpholino (Gene Tools, USA); TurboFect transfection reagent and Pierce™ protein A/G magnetic beads (Thermo Fisher Scientific, Germany); 2-

[3,6-Bis(acetyloxy)-2,7-dichloro-9H-xanthen-9-yl]benzoic acid (H2DCFDA, MedChemExpress, USA); Human FXII purified (Innovative Research, USA); Human Kininogen two chain purified high molecular weight (LOXO, Germany); recombinant mouse FXII (Abcam, USA).

Kits used in this study: RevertAid™ H minus first strand cDNA synthesis kit (Thermo Fisher Scientific, Germany); RNeasy Plus Micro Kit (Qiagen, Germany); AssayMax™ human FXII ELISA kit (EF1012-1; Assaypro LLC, USA); mouse D2D (D-Dimer) ELISA kit (orb775176, Biorbyt, UK); mouse albumin quantification ELISA kit (Bethyl Laboratories, Germany); Trichrome stain (Masson) kit and senescence cells histochemical staining kit (Sigma-Aldrich, Germany); DAB substrate kit (Vector Laboratories, USA); ScriptSeq v2 RNA-Seq library preparation kit (Epicentre Technologies, USA), TruSeq SBS Kit v3-HS (Illumina, USA); Olink® Target 48 Mouse (Olink Proteomics AB, Sweden).

FXII ELISA for human urine samples

For determination of urinary FXII, we used Human Factor XII ELISA Kit (Assaypro LLC, USA) which employs a quantitative sandwich enzyme immunoassay according to manufacturer's instructions. Human urine samples were centrifuged at 800 x g for 10 min to remove debris and dead cells and were diluted 1:2, then were added together with FXII standards to a polystyrene microplate coated with a murine antibody against human FXII and incubated for 2 h at room temperature. After washing the wells by washing buffer for 3 times, biotinylated human FXII antibody was added to each well and the plate was incubated for 1 h. Following washing (3 times using wash buffer), streptavidin-peroxidase conjugate was added and the plate was incubated for 30 min at room temperature. Plates were washed (3 times using wash buffer) and the chromogen substrate was added to each well and the plate was incubated for 30 min at room temperature for color development. Reaction was stopped by adding stop solution (as provided in the kit) and the absorbance was measured at 450 nm using a plate reader.

Albuminuria measurement for mice urine

Individual mice were kept in metabolic cages to collect 12 h urine samples at the indicated time points. Mouse urine albumin and creatinine were measured as previously described [1,2]. Urine albumin was determined using a mouse albumin ELISA kit (Mouse albumin ELISA quantification kit, Bethyl Laboratories, USA) according to the manufacturer's instructions. Urine creatinine was determined using a commercially available assay (Cobas c501 module, Roche Diagnostics, Basel, Switzerland) according to the manufacturer's instructions.

Determination of blood urea nitrogen (BUN)

Blood samples were obtained from the inferior vena cava and collected into tubes prefilled with sodium citrate (final concentration 3.8%). Plasma was obtained by centrifugation at 200g for 10 min. BUN was measured using a kinetic test kit with urease (Cobas c501 module; Roche Diagnostics, Basel, Switzerland).

D-Dimer ELISA

For determination of plasma D-Dimer levels, we used a mouse D2D(D-Dimer) ELISA kit (Biorbyt, UK) which employs a quantitative sandwich enzyme immunoassay according to manufacturer's instructions. Plasma samples (collected as described in the previous section) were diluted 1:5 in the assay diluent provided with the kit. Standards or samples were added to the appropriate microtiter plate wells which has been pre-

coated with an antibody specific to mouse D2D then with a biotin-conjugated antibody specific to mouse D2D. Next, avidin conjugated to HRP was added to each microplate well and incubated. After TMB substrate solution was added, the enzyme-substrate reaction was terminated by the addition of sulphuric acid solution and the color change was measured spectrophotometrically at a wavelength of 450nm ± 10nm. The concentration of mouse D2D in the samples was then determined by comparing the OD of the samples to the standard curve.

Immunoblotting

Kidney tissues and cell lysates were prepared using RIPA buffer containing 50 mM Tris (pH7.4), 1% NP-40, 0.25% sodium-deoxycholate, 150 mM NaCl, 1 mM EDTA, 1 mM Na_3VO_4, 1 mM NaF supplemented with protease and phosphatase inhibitor cocktails. Lysates were centrifuged (13000 g for 10 min at 4°C) and insoluble debris was discarded. Protein concentration in supernatants was quantified using BCA reagent. Equal amounts of protein were electrophoretically separated on 10% or 12.5% SDS polyacrylamide gel, transferred to PVDF membranes and probed with the desired primary antibodies at appropriate dilutions (KIM-1: 1:2000; Abcam-p21: 1:2000; CST-p21: 1:1000; γ-H2AX: 1:1000; SC-FXII: 1:500; Biorbyt-FXII: 1:1000; Biorbyt-uPAR: 1:1000; TS-uPAR: 1:1000; SC-integrin β1: 1:500; Kerafast-integrin β3: 1:1000; integrin α6: 1:500; integrin α5: 1:500; p-FAK: 1:1000; p-Src: 1:1000; Rqc1: 1:1000; NOX1: 1:1000; β-Actin: 1:1000; α-Tubulin: 1:4000). After overnight incubation with primary antibodies at 4°C, membranes were washed with TBS-T and incubated with anti-rabbit, anti-mouse or anti-rat horseradish peroxidase-conjugated IgG antibodies (1:4000) for 1 h at room temperature. Blots were developed with the enhanced chemiluminescence system. To compare and quantify levels of proteins, the density of each band was measured using ImageJ software. Equal loading for total cell or tissue lysates was determined by α-Tubulin immunoblots using the same blot whenever possible.

Immunoprecipitation

Protein lysates from HKC-8 cells subjected to the abovementioned interventions were used for immunoprecipitation. 1 µg of rabbit uPAR antibody (Cell Signaling Technology) was added to 500 µg protein in a total volume of 500 µl and were incubated with rotation for overnight at 4°C. Next day, 25 µL of Pierce™ Protein A/G magnetic beads (ThermoFischer Scientific) were added to the protein/antibody mixture and were incubated with rotation at room temperature for 2 h. The protein A/G beads were isolated using a magnetic rack and washed twice with TBST and once with ultra-pure water. Elution was achieved by boiling the samples in 1 x laemmli buffer for 10 min followed by beads separation.

Quantitative real time PCR (qRT-PCR)

Total RNA was extracted from kidney tissues or HKC-8 cells using RNeasy Plus Micro Kit (QIAGEN) following manufacturer's protocol. Quality of total RNA was ensured on agarose gel and by analysis of the A260/280 absorption ratio. The reverse transcription reaction was conducted using 1 µg of total RNA after treatment with DNase-I (5 U/5 µg RNA, 30 min, 37°C) followed by reverse transcription using RevertAid First Strand cDNA Synthesis Kit (ThermoFischer Scientific). qRT-PCR was carried out on Applied Biosystems ViiA 7™ Real-time system using SYBR green-Takyon polymerase mixture (Takyon™ Eurogentec) and gene specific primers (see Table S6). For quantitative analysis results were normalized to 18srRNA. Gene expression was analyzed using the $2^{-(\Delta\Delta Ct)}$ method. Reactions lacking cDNA served as negative controls.

RNA expression profiling and pathway analysis

RNA was isolated from mouse kidneys using RNeasy Plus Micro Kit (QIAGEN) following manufacturer's protocol. RNA was isolated from normoglycemic, age matched control mice and from mice with 24 weeks of persistent hyperglycemic following STZ injection as outlined above from WT and *F12*$^{-/-}$ mice. Quality and integrity of total RNA was verified using an Agilent Technologies 2100 Bioanalyzer (Agilent Technologies; Waldbronn, Germany).

For expression profiling by RNA sequencing (RNA-seq) a RNA sequencing library was generated from 500 ng total RNA. The Dynabeads® mRNA DIRECT™ Micro Purification Kit (Thermo Fisher) was used for purification of mRNA. The RNA sequencing library was generated using the ScriptSeq v2 RNA-Seq Library Preparation Kit (Epicentre) according to manufacturer's protocols. The libraries were sequenced on Illumina HiSeq2500 using TruSeq SBS Kit v3-HS (50 cycles, single ended run) with an average of 3 x107 reads per RNA sample. For each FASTQ file, a quality report was generated by FASTQC tool. Before alignment to reference genome, each sequence in the raw FASTQ files was trimmed on base call quality and sequencing adapter contamination using Trim Galore! wrapper tool. Reads shorter than 20 bp were removed from FASTQ files. Trimmed reads were aligned to the reference genome using open source short read aligner STAR (https://code.google.com/p/rna-star/) with settings according to log file. Feature counts were determined using R package "Rsubread". Only genes showing counts greater than 5 at least two times across all samples were considered for further analysis (data cleansing). Differential expression analysis was conducted in Rstudio using DEbrowser by the DEseq2 R package [3]. Following batch effect correction using the ComBat method, a threshold of FDR<0.05 and *P*<0.05 for each gene was used to screen differentially expressed genes (DEGs). DEGs heatmaps were generated using the online platform SRplot (http://www.bioinformatics.com.cn/srplot), with Z-score ((Gene expression value in sample of interest) - (Mean expression across all samples) / Standard Deviation)). Gene Set Enrichment Analysis software (GSEA; version 4.3.2), and ShinyGO 0.77 [4] were used for gene ontology and pathway enrichment analysis.

Immunohistochemistry

Kidney tissues of the mice were processed as previously described [5]. Mice were perfused with cold PBS and kidney tissues were incubated in 4% paraformaldehyde for 24 h before embedding them in paraffin for sectioning. Human or mouse paraffin kidney sections (5 µM thickness) were dewaxed, rehydrated in ethanol dilution series (99%, 80%, 70% 50% and 30%) then incubated with citrate-based antigen retrieval solution (H-3300, Vector laboratories) in a pressure cocker. Endogenous peroxidase activity was blocked by incubating the sections with 3% hydrogen peroxide solution for 30 min. Sections were then washed with PBS and blocked with PBS containing 3% donkey serum and 0.5% Triton X-100 for nuclear proteins or 0.05% tween-20 for cytoplasmic proteins for one h at room temperature. Sections were then incubated with primary antibodies against WT-1 (Abnova, 1:300); p21 (Abcam, 1:1000); FXII (Biorbyt, 1:200 or SC, 1:200), and uPAR (Biorbyt, 1:200 or R&D, 1:200) overnight at 4°C. Slides were washed with PBS and incubated with HRP-linked anti-mouse/rabbit or anti-goat IgG polymer (R&D) for 2 h at room temperature. Next, slides were washed and the colored product was developed using DAB substrate kit (SK-4100, Vector laboratories). The slides were then counterstained in hematoxylin Gill's for 1 min followed by washing in distilled water. The counter-stained sections were dehydrated in ethanol dilution series and mounted using ROTI®Histokitt II mounting medium (ROTH). The number of WT-1 positive cells was counted from at least 20 randomly selected glomeruli per mouse using ImageJ software. The percentage of p21 positive cells to the total cells in the field was calculated from 10 random images per mouse using ImageJ software.

Periodic acid-Shiff (PAS) Staining

Paraffin kidney sections were dewaxed and rehydrated in ethanol dilution series (99%, 80%, 70% 50% and 30%) before staining. PAS staining was performed by incubating the sections in 0.5% periodic acid for 5 min followed by washing with distilled water. Sections were then incubated in Schiff's reagent for 15 min followed by washing in running tap water for 10 min. Nuclear counter staining was performed by incubating the sections in hematoxylin Gill's for 1 min followed by washing in distilled water. The counter-stained sections were dehydrated in ethanol dilution series and mounted using ROTI®Histokitt II mounting medium (ROTH). The glomerular fractional mesangial area (FMA) was calculated following the current DCC (Diabetes Complications Consortium) protocol and as described before [1]. At least twenty glomeruli were investigated for each mouse. In every investigated glomerulus, the tuft glomerular area was determined using ImageJ software. FMA was calculated as the fractional area positive for PAS in the glomerular tuft area, reported as the percentage. For tubular injury score, ten visual fields per slide were randomly selected from the PAS stained sections. Tubular injury as defined by tubular dilation, loss of brush border, tubular atrophy, and cast formation was scored as percentage from the total number of tubuli observed in the field.

Masson's Trichrome staining (MTS)

Paraffin kidney sections were dewaxed and rehydrated in ethanol dilution series (99%, 80%, 70% 50% and 30%) then stained using the trichrome staining kit (HT15, Sigma) according to the manufacturer's instructions. Sections were dehydrated in ethanol dilution series and mounted using ROTI®Histokitt II mounting medium (ROTH). Renal tubulointerstitial fibrosis was evaluated as previously described [1]. Ten visual fields per slide were randomly selected. Interstitial fibrosis was quantified using ImageJ software, generating a binary image allowing automatic calculation of the stained area as percentage of the image area.

Senescence associated beta galactosidase staining

Senescence was evaluated using senescence cells histochemical staining kit (Sigma-Aldrich) according to the manufacturer's protocol. 5 µm thick cryro-sections of kidney tissues or PTCs grown on coverslips were washed in PBS and fixed for 1 min (2% formaldehyde, 0.2% glutaraldehyde, 7.04 mM Na_2HPO_4, 1.47 mM KH_2PO_4, 0.137 M NaCl, 2.68 mM KCl) and then rinsed in ice-cold PBS for 5 min. The cryo-sections or cells were incubated with freshly prepared X-gal staining solution (10 ml containing 0.25 ml of 40 mg/ml X-gal Solution, 125 µl of 400 mM potassium ferricyanide, 125 µl of 400 mM potassium ferrocyanide) at 37°C overnight. Next, excess staining solution was washed away using PBS, and the sections or cells were overlaid with 70% glycerol followed by staining assessment. For frozen sections, aqueous Eosin was used for counterstaining before mounting the slides with 70% glycerol. The development of blue dots/areas was counted as positive staining. Senescence was evaluated as the ratio (in percent) of the blue-colored area in each sample relative to the total area of at least 10 kidney cortical visual fields per section or 10 fields per coverslip.

Transmission electron microscopy (TEM)

Mouse kidneys were immersed in Karnovsky fixative solution (2.5% glutaraldehyde, 2.5% polyvidone 25, and 0.1 M sodium cacodylate, pH 7.4) overnight at 4°C. Next day, the tissues were washed with 0.1 M sodium cacodylate buffer, followed by post fixation in 0.1 M sodium cacodylate with 0.2% osmium tetroxide and 1.5% potassium ferrocyanide for 1 h. Tissues were washed with distilled water, contrasted *en bloc* with uranyl acetate, and finally dehydrated in graded ethanol series. The dehydrated tissues were

embedded in glycidyl ether 100-based resin. Reichert Ultracut S ultramicrotome (Leica Microsystems, Wetzlar, Germany) was used to cut ultrathin sections, followed by contrasting with uranyl acetate and lead citrate. Images of ultrastructure were acquired from EM 10 CR electron microscope (Carl Zeiss NTS, Oberkochen, Germany). Glomerular basement membrane (GBM) thickness was evaluated from 10 images per mouse using ImageJ software.

Immunofluorescence

Immunofluorescence staining of KIM-1, γH2A.X, Ki-67, Lamin B1, F4/80, FXII, uPAR, integrin β1, and HK was performed on paraffin embedded kidney sections or cells (HKC-8 and PTCs) grown on cover slips as previously described [5]. For paraffin sections, after dewaxing, rehydration and antigen retrieval, the sections were incubated with a blocking buffer (3% donkey serum in PBS with 0.5% Triton X-100 for nuclear proteins or 0.05% tween-20 for cytoplasmic proteins) followed by overnight incubation with primary antibodies. For cover slips, after fixation in 3.7% paraformaldehyde, the cover slips were blocked (1% BSA in PBS) followed by overnight incubation with primary antibodies. Sections or coverslips were washed then incubated with secondary antibodies for 2 h followed by further washing and mounting with Vectashield mounting medium containing the nuclear stain DAPI. Images were acquired using Leica THUNDER imaging microscope (DMi8 platform; Leica, Germany). Images were analyzed using Leica Application Suite X software 8, (LAS X; Leica, Germany). Fluorescence intensities were analyzed using image-J software according to the method of corrected total cell fluorescence (CTCF) as outlined previously [6].

Proximity ligation assay (PLA)

PLA experiments were performed on cells grown on coverslips (HKC-8 and PTCs) or on murine and human paraffin kidney sections using Duolink® in situ detection reagents kit (Sigma-Aldrich) according to manufacturer's instructions and as described previously [7]. Cells on cover slips were fixed in 3.7% paraformaldehyde and kidney sections were dewaxed, rehydrated and subjected to antigen retrieval. Cells and kidney sections were then blocked in Duolink® blocking Solution in a humidity chamber for 60 min at 37°C. Cells or sections were incubated overnight at 4°C with primary antibodies. For HKC-8 cells, the following antibodies were used: rabbit anti-uPAR (1:200; Biorbyt) and mouse anti-Integrin β1 (1:200, clone 12G10; Santa Cruz). For PTC, the following antibodies were used: rabbit anti-FXII (1:200; Biorbyt) and goat anti-uPAR (1:200; R&D systems). For murine kidney tissues, the following antibodies were used: goat anti-uPAR (1:200; R&D systems) and rat anti-Integrin β1 (1:100, clone 9EG7; BD Biosciences, generated using Duolink® in situ probemaker plus). For human kidney tissues, the following antibodies were used: goat anti-uPAR (1:200; R&D systems) and mouse anti-Integrin β1 (1:200, clone 12G10; Santa Cruz). The primary antibodies were diluted in Duolink® antibody diluent. The following day, cells and sections were washed twice with Duolink® wash buffer A and incubated in 1x PLA probes (with a unique short DNA attached strand) in Duolink® antibody diluent (HKC-8 cells: mouse minus and rabbit plus probes; PTC: goat minus and rabbit plus probes; murine sections: goat minus probe; human sections: mouse minus and goat plus probes) at 37°C for 1 h. Cells and kidney sections were then washed twice with Duolink® wash buffer A and treated with ligase in 1x ligation buffer for 30 min at 37°C to link the probe-attached oligonucleotides, followed by washing twice with Duolink® wash buffer A and treatment with polymerase in 1x amplification buffer for 100 min at 37°C to amplify the DNA using fluorescently labeled complementary oligonucleotides. Finally, cells and tissues were washed twice in 1X Duolink® wash buffer B at room temperature for 10 min, followed by 1 min of washing in 0.01x Duolink® wash buffer B. Following the washing step, cells were incubated with Alexa Fluor™ 488 Phalloidin (1:300) for 1 h at room temperature. Cells and sections were

mounted using Duolink® in situ mounting medium with DAPI. Images were acquired using Leica THUNDER imaging microscope (5 fields per kidney section and a minimum of 30 cells per coverslip). The number of red PLA dots was quantified using ImageJ and the analyze particles plugin.

Detection of ROS in living cells

At the end of FXII exposure for different time points, the medium was removed from HKC-8 cells and they were washed once with PBS, followed by incubation with 0.5 µM of the cell permeable ROS indicator 2-[3,6-Bis(acetyloxy)-2,7-dichloro-9H-xanthen-9-yl] benzoic acid (H2DCFDA) dissolved in PBS in the dark for 30 min at 37°C. Following incubation, cells were washed once with PBS and supplemented with fresh culture medium. Green fluorescence of oxidized H2DCFDA was monitored using microscopy, and the intensity of the fluorescence was analyzed using image-J software according to the method of corrected total cell fluorescence (CTCF).

Staining for oxidative DNA damage

Immunofluorescence staining of 8-O-dG was performed on paraffin embedded kidney sections or cells (HKC-8 and PTCs) grown on cover slips as previously described [8]. Dewaxed and rehydrated kidney sections were treated with proteinase K for 30 min at 37°C. The DNA was denatured using 2N HCl for 5 min at room temperature followed by neutralization with 1M Tris-base for 5min at room temperature. Sections were blocked in 3% donkey serum in PBS. Cells on cover slips were fixed in 1:1 methanol, acetone for 20 min at -20°C. Cells were treated with 0.05N HCl for 5 min on ice followed by washing in PBS. DNA was denatured using 0.15N NaOH in 70% ethanol for 4 min followed by washing in PBS. Cells were incubated with proteinase K for 10 min at 37°C followed by blocking in 1% BSA in PBS. Sections and cells were incubated with the primary antibody against 8-O-dG (1:250) for overnight at 4°C. Cells were washed and incubated with the appropriate secondary antibody for 2 h at room temperature followed by washing and mounting with Vectashield mounting medium containing the nuclear stain DAPI. Images were acquired using Leica THUNDER imaging microscope (DMi8 platform; Leica, Germany). Images were analyzed using Leica Application Suite X software 8 (LAS X; Leica, Germany). Fluorescence intensities were analyzed using image-J software according to the method of corrected total cell fluorescence (CTCF).

Protein simulation using Alphafold 2

Prediction of the uPAR-FXII multimer was performed using ColabFold (alphafold2_multimer_v3, https://colab.research.google.com/github/sokrypton/ColabFold/blob/main/AlphaFold2.ipynb) with default parameters. The resulting structures were clustered based on pairwise RMSD calculations. Within each cluster, the structure with the highest ipTM score was selected for further analysis. Alphafold 2 showed high confidence scores and convergence for each individual chain, but lower confidence and convergence for the inter-peptide arrangement of the two chains. To identify biologically meaningful configurations, the PPI3D website (http://bioinformatics.ibt.lt/ppi3d/) was used to find experimental structures of proteins that had similar interaction patterns to the candidates. In addition, ConSurf (https://consurf.tau.ac.il/consurf_index.php) was used to identify highly conserved residues that were prioritized in the interaction search. Finally, the PDB file 2HPP was identified as having a remarkably similar binding mode to one of the predicted models. In both structures, the Kringle domain (also present in FXII) showed binding interactions with another protein.

Peptide synthesis

Sequential peptides to fibronectin type II domain (HR13: HRQLYHKCTHKGR) and kringle domain (TY10: TYRNVTAEQA, and PW15: PWCFVLNRDRLSWEY) of the heavy chain of human FXII in addition to the peptide PGS20 of the domain 2 of the human uPAR (PGSNGFHNNDTFHFLKCCNT) [9] were synthesized at the Core Unit Peptid-Technologien (Medical Faculty, Leipzig University, Germany). Solid phase peptide synthesis was utilized on an automated peptide synthesizer MultiPep (Intavis AG, Köln, Germany) using standard Fmoc-chemistry. Fmoc-amino acids were coupled using N,N-diisopropylethylamine (6 equiv.) and O-(7-azobenzotriazol-1-yl)-1,1,3,3-tetramethyluroniumhexafluorophosphate (3 equiv.) in DMF for 2 h. The N-terminal Fmoc protecting group was removed by washing the resin with 20% piperidine for 20 min. The final deprotection from the resin was done in TFA/TIS/water (95:2.5:2.5) for 3 h at room temperature. The FXII peptides were named for the first 2 amino acids followed by the number of the residues in the peptide. Peptide sequences are listed in Table S5.

Peptides FXII-618 [10] and FXII-900 [11] were synthesized at the Institute for Drug Discovery (Medical Faculty, Leipzig University, Germany). Solid phase peptide synthesis was utilized on an automated peptide synthesizer CEM MultiPep 1 (CEM, Kamp-Lintfort) using Fmoc-chemistry. Fmoc-amino acids were coupled using 4.1 equiv. Fmoc-amino acid, 4 equiv. N,N'-Diisopropylcarbodiimide, and 4 equiv. Oxyma. The N-terminal Fmoc protecting group was removed by adding 20 % piperidine in DMF to resin (35 equiv.). After each coupling step, resin was capped with 7.35 equiv. acetic anhydride in DMF for 5 min. The final deprotection and cleavage from resin was done in TFA/TIS/water/1,2-Ethandithiol (92.5:2.5:2.5:2.5) for 4 h at room temperature. Peptides were further cyclized by adding 1.2 equiv. 1,3,5-triacryloyl-1,3,5-triazine to a 1 mM peptide solution in NH_4CO_3 buffer/acetonitrile (70:30) at pH 8.

Peptide stability

An aliquot of 10 µL PW15 stock solution (80 mM in H_2O/ACN (1:1 v/v)) was added to 1590 µL medium to achieve a final peptide concentration of 500 µM. The mixture was incubated at 37°C. At certain time points (0 min, 2 min, 5 min, 7 min, 10 min, 15 min, 20 min, 30 min, 45 min, 1 h, 1.5 h, 2 h, 3 h, 4 h, 5 h, 6 h, 7 h, 20 h, 24 h) aliquots of 20 µL were taken from the mixture and incubated with 12 M urea solution at room temperature for 30 min for protein denaturation. Afterwards, 200 µL of isopropanol were added and the samples were incubated at -22°C for at least 30 min to precipitate medium components. The samples were centrifuged (4°C, 9000 x g, 45 min) and the supernatants evaporated to dryness in a vacuum centrifuge. The residual pellet was dissolved in 40 µL of 60% ACN in H_2O. With these samples HPLC and LCMS measurements were performed on a SHIMADZU LCMS-2020 with a linear gradient of 5-95% ACN in H_2O over 10 min on an ACE 3 C18-300 column (flow: 0.35ml/min, injection volume: 25 µL for HPLC, 5 µL for LCMS). For each time point three replicates were measured as well as a control series with the peptide in water. PW15 eluted at 7.85 min. Chromatograms were measured for UV absorption at 215 nm. The respective signals were integrated and the peak areas were monitored over time to determine the peptides stability in the medium.

uPAR surface staining by flow cytometry

HKC-8 cells were treated with purified human FXII (62 nM) in the presence of 10µ M of Zn^{2+} for 6, 24 and 48 h. Cells were washed from the culture medium with PBS and harvested then fixed in 3.7% paraformaldehyde for 15 min at room temperature without permeabilization. Cells were washed with PBS and pelleted down, then stained with rabbit anti-uPAR (1:200; Biorbyt) prepared in 1% BSA in PBS for 1 h at room temperature. Cells were then washed in PBS and stained with DyLight™ 649 donkey anti-rabbit

IgG (1:500; Biolegend) for 1 h at room temperature. Cells were washed in PBS, pelleted down and resuspended in 1% BSA in PBS and the mean fluorescence intensity (MFI) of uPAR surface expression was acquired using flow cytometry (Attune NxT; ThermoFischer Scientific), and data were analyzed with FlowJo V.10 software (BD Biosciences).

Cytokine assay using Olink® Target 48 Mouse

Selected SASP-related cytokines and chemokines were analyzed in kidney tissue lysates using the Olink® Target 48 Mouse kit according to manufacturer's instruction. Kidney tissue lysates were prepared in RIPA buffer at a final concentration of 0.5 mg/ml. 1 µL of each sample was added into the appropriate wells of the provided plate followed by addition of 3 µL of the detection mix (incubation plate). The plate was sealed and incubated for overnight at 4°C. After incubation, 96 µL of the extension mix were added to each well and the incubation plate was placed in a thermal cycler [50 °C for 20 min, 95 °C for 5 min (95 °C for 30 sec; 54 °C for 1 min; 60 °C for 1 min) x 17 cycles]. Detection was conducted using Olink® 48.48 IFC for protein expression. IFC was primed on Olink® Signature Q100. 46 µl of detection mix was added to the primer plate. 7.2 µl of the detection mix were transferred in a new 96-well plate (sample plate) by reverse pipetting. 2.8 µl was transferred from incubation plate to the sample plate by forward pipetting. 5 µl from the primer plate and sample plate respectively were added into the primed 48.48 IFC left and right inlets. The IFC was then loaded and run on Olink® Signature Q100. The data were analyzed using the Olink® NPX™ Signature software.

Cell cycle analysis

PTCs were treated with recombinant mouse FXII (62 nM) in the presence of 10µ M of Zn^{2+} for 6, 24 and 48 h. Cells were washed from the culture medium with PBS and harvested then fixed in ice cold 70% ethanol for 30 min at 4°C. Cells were washed twice in PBS and pelleted down, then the pellet was resuspended in 500 µl of FxCycle™ promidium iodide (PI)/RNase staining solution (ThermoFischer Scientific) for 30 min at room temperature. After incubation, the DNA content was directly analyzed without washing using flow cytometry (Attune NxT; ThermoFischer Scientific), and data were analyzed with FlowJo V.10 software (BD Biosciences).

Supplementary tables

Characteristic	C (n=6)	DKD (n=5)
**Age** (years)	71.5 ± 3.1	78.2 ± 2.5
**Sex** (female/male)	(3/3)	(2/3)
**History (n) of**		
Hypertension	2	4
Brain tumor	1	-
COPD	1	-
Steatosis hepatitis	1	-
peripheral arterial occlusive disease	1	-
Hypothyroidism	1	-
Type 2 diabetes	-	5
**Diabetic complications (n)**		
Diabetic neuropathy	-	-
Diabetic kidney disease (DKD)	-	5
**Serum creatinine** (mg/dl)	1.1 ± 0.17	1.9 ± 0.36 (p=0.0327)
**Serum urea** (mg/dl)	6.6 ± 0.66	10.8 ± 1.78 (p=0.0337)

Table S1. Anthropometric, clinical and metabolic characteristics of kidney biopsies of control and diabetic kidney disease patients. Data represents mean±SEM. Comparison by two-tailed unpaired student's t-test. Abbreviations: C: Control, DKD: Diabetic Kidney Disease, COPD: chronic obstructive pulmonary disease.

Characteristic	Control	Low risk	Moderate risk	High risk	Very high risk
**Number of patients**	**n=57**	**n=45**	**n=53**	**n=29**	**n=18**
**Age** (years)	63.10 ± 0.34	71.0±0.04 (p<0.0001)	70.1±0.7 (p<0.0001)	71.03±0.9 (p<0.0001)	74.1±1.05 (p<0.0001)
**Sex** (female/male)	(24/33)	(5/40)	(15/38)	(11/18)	(5/13)
**BMI** (kg/m²)	28.44±0.81	30.01±0.73	30.56±0.61 (p=0.0251)	30.65±0.8 (p=0.0610)	31.2±1.04 (p=0.0408)
**Diabetes therapy (N)**					
Metformin	-	28	37	18	5
DPP-4	-	8	17	7	6
Glinide	-	6	7	7	4
Long-acting insulin	-	8	14	11	12
**Other medication (N)**					
RAAS-inhibitors	6	37	41	20	18
Beta-blockers	5	23	27	15	12
Thiazide diuretics	0	8	0	0	0
Loop diuretics	0	2	0	0	0
Calcium-antagonist	0	9	19	20	6
Statin	1	27	23	14	7
Ezetimib	0	2	0	0	0
Acetyl-salicylic acid	1	17	0	0	0
L-Thyroxin	3	7	11	3	1
**Glycemic control**					
HbA1c (%)	5.43±0.11	6.11±0.1 (p<0.0001)	6.4±0.9 (p<0.0001)	6.2±0.21 (p=0.0002)	6.10±0.13 (p<0.0001)
**Serum creatinine** (mg/dl)	1.05±0.09	0.91±0.02	0.95±0.02	1.05±0.05	1.50±0.07 (p=0.0027)
**eGFR** (ml/min/1.73m²)	97.98±0.25	82.98±1.01 (p<0.0001)	68.9±1.4 (p<0.0001)	73.8±3.2 (p<0.0001)	40.9±3.0 (p<0.0001)
**Albuminuria** (mg albumin/g urine-creatinine)	8.28±0.9	11.29±1.11 (p=0.0211)	85.4±10.4 (p<0.0001)	730.8±203.8 (p<0.0001)	802 ±176.2 (p<0.0001)

Table S2. Anthropometric, clinical and metabolic characteristics of non-diabetic controls and type-2 diabetic individuals from the LIFE-adult cohort. The severity of DKD was classified according to the KDIGO criteria as low, moderate, high and very high risk of chronic kidney disease. Data represents mean±SEM, comparison by two-tailed Mann–Whitney test (each diabetic group against controls). Abbreviations: BMI: body mass index, DPP-4: dipeptydil peptidase 4, RAAS: renin-angiotensin-aldosterone system, eGFR: estimated glomerular filtration rate.

Characteristic	Control	Low risk	Moderate risk	High risk	Very high risk
**Number of patients**	**n=35**	**n=61**	**n=57**	**n=17**	**n=5**
**Age** (years)	55.91 ± 1.58	68.65±0.99 (p<0.0001)	64.11±1.6 (p<0.0001)	58±3.88	65±6.34
**Sex** (female/male)	(7/28)	(39/22)	(33/24)	(6/11)	(3/2)
**BMI** (kg/m²)	26.50±0.81	29.52±0.64 (p<0.0044)	31.21±0.80 (p<0.0001)	33.94±1.55 (p<0.0001)	28.24±3.89
**Diabetes therapy (N)**					
Metformin	-	27	25	6	2
Oral antidiabetics	-	30	24	5	3
Long-acting insulin	-	19	25	10	3
**Other medication (N)**					
RAAS-inhibitors	5	37	32	12	2
Beta-blockers	5	21	29	7	2
Thiazide diuretics	1	15	15	4	0
Loop diuretics	0	7	6	4	1
Calcium-antagonist	1	14	10	2	2
Statin	3	26	23	9	3
Spironolactone	1	2	4	1	0
Acetyl-salicylic acid	3	16	18	6	3
Clopidogrel	0	1	2	0	0
**Glycemic control**					
HbA1c (%)	5.43±0.05	6.88±0.15 (p<0.0001)	7.07±0.15 (p<0.0001)	7.5±0.28 (p<0.0001)	8.06±1 (p=0.0259)
**eGFR** (ml/min/1.73m²)	94.89±1.62	75.67±1.08 (p<0.0001)	81.97±2.93 (p<0.0001)	72.47±7 (p=0.0067)	27.68±7.67 (p<0.0001)
**Albuminuria** (mg albumin/g urine-creatinine)	9.42±1.23	7.96±0.81	70.33±9.3 (p<0.0001)	1129.22±417.7 (p<0.0001)	130.19±97.83 (p=0.0388)

Table S3. Anthropometric, clinical and metabolic characteristics of non-diabetic controls and type-2 diabetic individuals from the HEIST-DiC cohort. The severity of DKD was classified according to the KDIGO criteria as low, moderate, high and very high risk of chronic kidney disease. Data represents mean±SEM, comparison by two-tailed Mann–Whitney test (each diabetic group against controls). Abbreviations: BMI: body mass index, RAAS: renin-angiotensin-aldosterone system, eGFR: estimated glomerular filtration rate.

Interacting residues in FXII	
number	amino acid
53	TYR
54	HIS
55	ARG
56	GLN
58	TYR
59	HIS
60	LYS
62	THR
63	HIS
64	LYS
65	GLY
66	ARG
79	PHE
83	GLN
221	ARG
223	LEU
244	GLU
247	TYR
248	ARG
249	ASN
250	VAL
251	THR
254	GLN
258	TRP
269	PRO
270	ASP
271	ASN
272	ASP
273	ILE
276	TRP
278	PHE
282	ARG
283	ASP
284	ARG
285	LEU
286	SER
287	TRP
289	TYR

Interacting residues in uPAR	
number	amino acid
54	TRP
55	GLU
56	GLU
57	GLY
59	GLU
80	ARG
81	THR
82	GLY
83	LEU
84	LYS
85	ILE
108	THR
109	TYR
111	ARG
112	SER
113	ARG
116	GLU
118	ILE
123	SER
124	ASP
125	MET
128	GLU
129	ARG
130	GLY
131	ARG
132	HIS
133	GLN
134	SER
135	LEU
136	GLN
150	HIS
151	TRP
152	ILE
153	GLN
154	GLU
155	GLY
157	GLU
158	GLY
159	ARG
161	LYS
164	ARG
196	THR
197	LYS
207	GLU

Table S4. Interacting residues in FXII and uPAR. The interacting residues in fibronectin type II (green) and Kringle (yellow) domains of FXII, and in domains 1 (blue) and 2 (pink) of uPAR as predicted by AlphaFold2_multimer_v3 computational modeling.

Peptide name	Peptide sequence	Targeted domain
HR13	HRQLYHKCTHKGR	FXII's Fibronectin type-II (H54-R66)
TY10	TYRNVTAEQA	FXII's Kringle (T246-A255)
PW15	PWCFVLNRDRLSWEY	FXII's Kringle (P275-Y289)
RL20	RLWEEGEELELVEKSCTHSE	uPAR domain 1 (R52-E71)
DL19	DLCNQGNSGRAVTYSRSRY	uPAR domain 1 (D96-Y114)
DV20	DVVTHWIQEGEEGRPKDDRH	uPAR domain 2 (D146-H165)
PGS20 [9]	PGSNGFHNNDTFHFLKCCNT	uPAR domain 2 (P176-T195)
FXII-618 [10]	RCFRLPCRQLRCR (cyclized via reaction of Cys-thiols with 1,3,5-triacryloyl-1,3,5-triazine)	FXIIa
FXII-900 [11]	rsCF4FRLPCHQLRβhC (cyclized via reaction of Cys-thiols with 1,3,5-triacryloyl-1,3,5-triazine)	FXIIa

Table S5. Peptide sequences. The sequences of the synthetic sequential peptides for FXII's Fibronectin type-II and Kringle domains, the uPAR domains 1 and 2 and the cyclic FXII peptide inhibitors.

Target	Forward primer sequence (5'-3')	Reverse primer sequence (5'-3')
m-*F12*	TGGGGTCACCAGTTCGAG	CGATCCAGGGCGATAAAG
m-*Cdkn1a*	TCCACAGCGATATCCAGACA	GGACATCACCAGGATTGGAC
m-*Cdkn2a*	TGTTGAGGCTAGAGAGGATCTTG	CGAATCTGCACCGTAGTTGAGC
m-*Il-6*	CGTGGAAATGAGAAAAGAGTTGT	GGTAGCATCCATCATTTCTTTGT
m-*Ccl8*	CATGGAAGCTGTGGTTTTCCAGA	CCATGTACTCACTGACCCACTTC
m-*Ccl11*	TCCATCCCAACTTCCTGCTGCT	CTCTTTGCCCAACCTGGTCTTG
m-*Cxcl2*	GGCGGTCAAAAAGTTTGCCTT	CAGTTAGCCTTGCCTTTGTTCAG
m-*Cxcl13*	CATAGATCGGATTCAAGTTACGCC	GTAACCATTTGGCACGAGGATTC
m-*Cxcl14*	TACCCACACTGCGAGGAGAAGA	CGCTTCTCGTTCCAGGCATTGT
m-*Icam-1*	AGTCCGCTGTGCTTTGAGA	CACACTCTCCGGAAACGAATACA
m-*Mmp-3*	CTCCACAGACTTGTCCCGTT	ATGCTGTGGGAGTTCCATAGAG
m-*Tnf-a*	GGTGCCTATGTCTCAGCCTCTT	GCCATAGAACTGATGAGAGGGAG
m-*Grem-2*	CTCGCCTTACAAGGATGGTAGC	AGGTACTTGCGCTCGGTGACTA
m-*Lox*	CATCGGACTTCTTACCAAGCCG	GGCATCAAGCAGGTCATAGTGG
m-*Tgfb*	GCTGAACCAAGGAGACGGAA	GGGCTGATCCCGTTGATTTC
m-*Itgb1*	TCAGACTTCCGCATTGGCTTT	TCTTAGCTTTGCTGGGGTTGT
m-*Itgb3*	CAAGTGGGACACAGCAAACAA	CATTAAGTCCCCCGGTAGGTG
m-*Itgb4*	GCTCCTGCGTACAGTGCC	ACTCCACCACCTCTTCTTTCTT
m-*Itga6*	GTTTTTACTGTGGAAGTGTGGCT	GGATCTCAGCCTTGTGATAGGT
m-*Itgam*	GCCATTGTGGCCAGTGAGAA	ATGGCGTACTTCACAGGCAG
m-*Itgav*	TGAAGGCGCAGAATCAAGGG	GTCTTGCTAAGGCTTCGTTGT
m-Klkb1	TGTCGGACCATCTGCACCTATC	GGAGTAGAGGAACTTGGTGTGC
m-Kng1	GCCAACTTCTCACAGAGCTGTAC	TGACCAAGCACCTCCTTCAGCT
m-F11	AGGTGACTCTGCACATCAGCCA	ACAATGCCACCGTAGACACGCA
18srRNA	GGCCCTGTAATTGGAATGAGTC	CCAAGATCCAACTACGAGCTT
h-*Cdkn1a*	GTCAGTTCCTTGTGGAGCCG	ACATGGCGCCTCCTCTG
h-*Cdkn2a*	GGAGGCCGATCCAGGTCA	TGAGAGTGGCGGGGTCG
h-*Il-6*	CCCACCGGGAACGAAAGAG	GGACCGAAGGCGCTTGT
h-*Ccl2*	TCGCTCAGCCAGATGCAA	CTGCACTGAGATCTTCCTATTGGT
h-*Ccl8*	CAAGATGAAGGTTTCTGCAGCG	TTGGAATGGAAACTGAATCTGGCT
h-*Icam-1*	GGTAGCAGCCGCAGTCATAA	TCCCTTTTTGGGCCTGTTGT
h-*Mmp3*	TCACTCACTCACAGACCTGAC	GAGTCAGGGGGAGGTCCATAG
h-*Tgfb*	TTGAGCCGTGGAGGGGAAAT	GGCCGGTAGTGAACCCG

Table S6. Primer sequences. Sequences of primers used for qRT-PCR in the current study. Abbreviations: m: mouse, h: human.

Supplementary references

1. Marquardt, A., *et al.* Farnesoid X Receptor Agonism Protects against Diabetic Tubulopathy: Potential Add-On Therapy for Diabetic Nephropathy. *Journal of the American Society of Nephrology : JASN* (2017).
2. Shahzad, K., *et al.* Podocyte-specific Nlrp3 inflammasome activation promotes diabetic kidney disease. *Kidney international* **102**, 766-779 (2022).
3. Love, M.I., Huber, W. & Anders, S. Moderated estimation of fold change and dispersion for RNA-seq data with DESeq2. *Genome biology* **15**, 550 (2014).
4. Ge, S.X., Jung, D. & Yao, R. ShinyGO: a graphical gene-set enrichment tool for animals and plants. *Bioinformatics* **36**, 2628-2629 (2020).
5. Madhusudhan, T., *et al.* Signal integration at the PI3K-p85-XBP1 hub endows coagulation protease activated protein C with insulin-like function. *Blood* **130**, 1445-1455 (2017).
6. McCloy, R.A., *et al.* Partial inhibition of Cdk1 in G 2 phase overrides the SAC and decouples mitotic events. *Cell cycle* **13**, 1400-1412 (2014).
7. Madhusudhan, T., *et al.* Defective podocyte insulin signalling through p85-XBP1 promotes ATF6-dependent maladaptive ER-stress response in diabetic nephropathy. *Nature communications* **6**, 6496 (2015).
8. Soultanakis, R.P., *et al.* Fluorescence detection of 8-oxoguanine in nuclear and mitochondrial DNA of cultured cells using a recombinant Fab and confocal scanning laser microscopy. *Free radical biology & medicine* **28**, 987-998 (2000).
9. LaRusch, G.A., *et al.* Factor XII stimulates ERK1/2 and Akt through uPAR, integrins, and the EGFR to initiate angiogenesis. *Blood* **115**, 5111-5120 (2010).
10. Baeriswyl, V., *et al.* A Synthetic Factor XIIa Inhibitor Blocks Selectively Intrinsic Coagulation Initiation. *ACS chemical biology* **10**, 1861-1870 (2015).
11. Wilbs, J., *et al.* Cyclic peptide FXII inhibitor provides safe anticoagulation in a thrombosis model and in artificial lungs. *Nature communications* **11**, 3890 (2020).

Fig. S.1

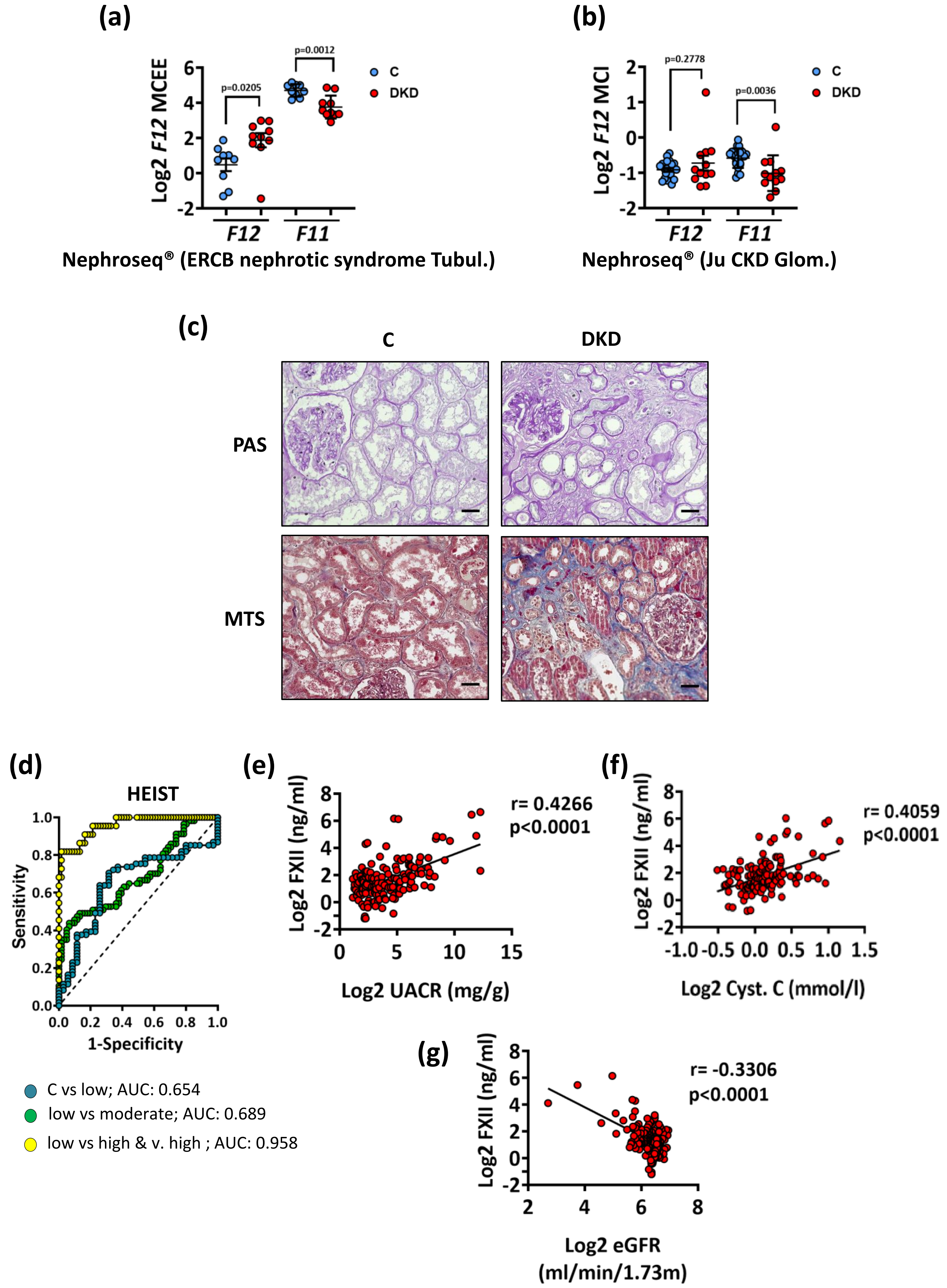
(a)
p=0.0205
p=0.0012
Log2 F12 MCEE
6
4
2
0
-2
F12
F11
C
DKD
Nephroseq® (ERCB nephrotic syndrome Tubul.)
(b)
p=0.2778
p=0.0036
Log2 F12 MCI
2
1
0
-1
-2
F12
F11
C
DKD
Nephroseq® (Ju CKD Glom.)
(c)
C
DKD
PAS
MTS
(d)
HEIST
Sensitivity
1.0
0.8
0.6
0.4
0.2
0.0
0.0 0.2 0.4 0.6 0.8 1.0
1-Specificity
C vs low; AUC: 0.654
low vs moderate; AUC: 0.689
low vs high & v. high ; AUC: 0.958
(e)
Log2 FXII (ng/ml)
8
6
4
2
0
-2
0 5 10 15
Log2 UACR (mg/g)
r= 0.4266
p<0.0001
(f)
Log2 FXII (ng/ml)
8
6
4
2
0
-2
-1.0 -0.5 0.0 0.5 1.0 1.5
Log2 Cyst. C (mmol/l)
r= 0.4059
p<0.0001
(g)
Log2 FXII (ng/ml)
8
6
4
2
0
-2
2 4 6 8
Log2 eGFR
(ml/min/1.73m)
r= -0.3306
p<0.0001

Fig. S1 Upregulation of FXII in human DKD

a,b) Dot plots summarizing *F12* and *F11* expression in the tubulointerstitial (a) and glomerular (b) compartments comparing diabetic kidney disease patients (DKD) to healthy controls (C) (ERCB nephrotic syndrome and the Ju CKD datasets of the Nephroseq® database, respectively). Dot plots reflecting mean±SEM; two-tailed unpaired student's t-test comparing C and DKD. MCEE: median-centered expression estimate; MCI: median centered intensity.

c) Exemplary histological images of periodic acid Schiff staining (top panel, PAS) and interstitial fibrosis (bottom panel, Masson's trichrome stain, MTS) in human kidney sections of non-diabetic controls (C) or diabetic patients with DKD. Scale bars represent 20 µm.

d) Receiver operating characteristic (ROC) analyses of urinary FXII (ng/ml; ELISA) in diabetic individuals with low risk of CKD compared to non-diabetic controls (blue) or in diabetic individuals with moderate risk of CKD (green), or high and very high risk (yellow) compared to low risk patients in the HEIST-DiC cohort. AUC: area under the curve.

e,f) Line graphs representing the positive correlation of urinary FXII (ng/ml) with urinary albumin creatinine ration (UACR; mg albumin/g creatinine; e, n=168) and the negative correlation with the estimated glomerular filtration rate (eGFR, ml/min/1.73m²; f, n=158) in diabetic individuals from the HEIST-DiC cohort. Confidence interval of r (Pearson coefficient) and *P* values (two-tailed) were calculated by linear regression.

g) Line graph representing the positive correlation of urinary FXII (ng/ml) with serum cystatin C levels (Cyst. C; mmol/l, n=142) in diabetic individuals from the LIFE-ADULT cohort. Confidence interval of r (Pearson coefficient) and *P* values were calculated by linear regression.

Fig. S.2

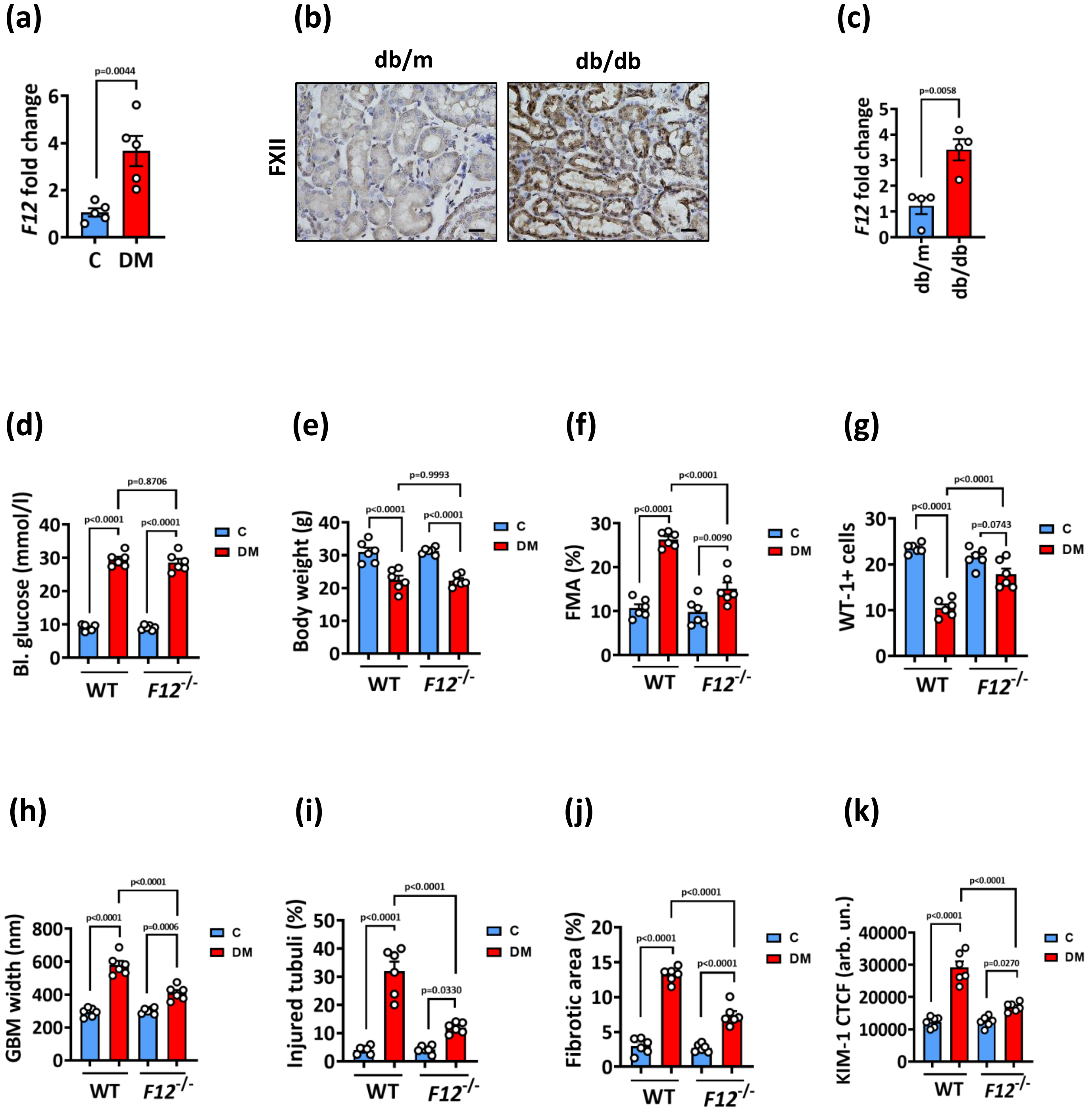
(a)
p=0.0044
F12 fold change
C
DM
(b)
db/m
db/db
FXII
(c)
p=0.0058
F12 fold change
db/m
db/db
(d)
p=0.8706
p<0.0001
p<0.0001
Bl. glucose (mmol/l)
WT
F12-/-
C
DM
(e)
p=0.9993
p<0.0001
p<0.0001
Body weight (g)
WT
F12-/-
C
DM
(f)
p<0.0001
p<0.0001
p=0.0090
FMA (%)
WT
F12-/-
C
DM
(g)
p<0.0001
p<0.0001
p=0.0743
WT-1+ cells
WT
F12-/-
C
DM
(h)
p<0.0001
p<0.0001
p=0.0006
GBM width (nm)
WT
F12-/-
C
DM
(i)
p<0.0001
p<0.0001
p=0.0330
Injured tubuli (%)
WT
F12-/-
C
DM
(j)
p<0.0001
p<0.0001
p<0.0001
Fibrotic area (%)
WT
F12-/-
C
DM
(k)
p<0.0001
p<0.0001
p=0.0270
KIM-1 CTCF (arb. un.)
WT
F12-/-
C
DM

Fig. S2 FXII correlates with histopathology in murine DKD

a) Dot plot summarizing kidney *F12* expression (qRT-PCR) comparing normoglycemic controls (C) and hyperglycemic (DM) WT mice. Dot plot reflecting mean±SEM of 5 mice per group; two-tailed unpaired student's t-test.

b) Exemplary histological images of mouse kidney sections stained for FXII comparing non-diabetic controls (db/m) and diabetic mice (db/db, type-2 diabetes model). FXII is detected by HRP-DAB reaction (brown); hematoxylin nuclear counter stain (blue). Scale bars represent 20 µm. Representative images of 4 mice per group.

c) Dot plot summarizing kidney *F12* expression (qRT-PCR) in experimental groups (as described in b). Dot plot reflecting mean±SEM of 4 mice per group; two-tailed unpaired student's t-test.

d,e) Dot plots summarizing average blood glucose (mmol/l; d) and body weight (g; e) comparing normoglycemic controls (C) and hyperglycemic (DM) WT and $F12^{-/-}$ mice. Dot plots reflecting mean±SEM of 6 mice per group; two-way ANOVA with Tukeys's multiple comparison test.

f-k) Dot plots summarizing quantification of fractional mesangial area (FMA; f), Wilm's tumor 1 positive cells (WT-1; g), glomerular basement membrane width (nm, GMB; h), percentage of injured tubuli (i), fibrotic area (j), and KIM-1 intensity (k) in experimental groups (as described in d). Dot plots reflecting mean±SEM of 6 mice per group; two-way ANOVA with Tukeys's multiple comparison test. CTCF: corrected total cell fluorescence. Arb. un.: arbitrary units.

Fig. S.3

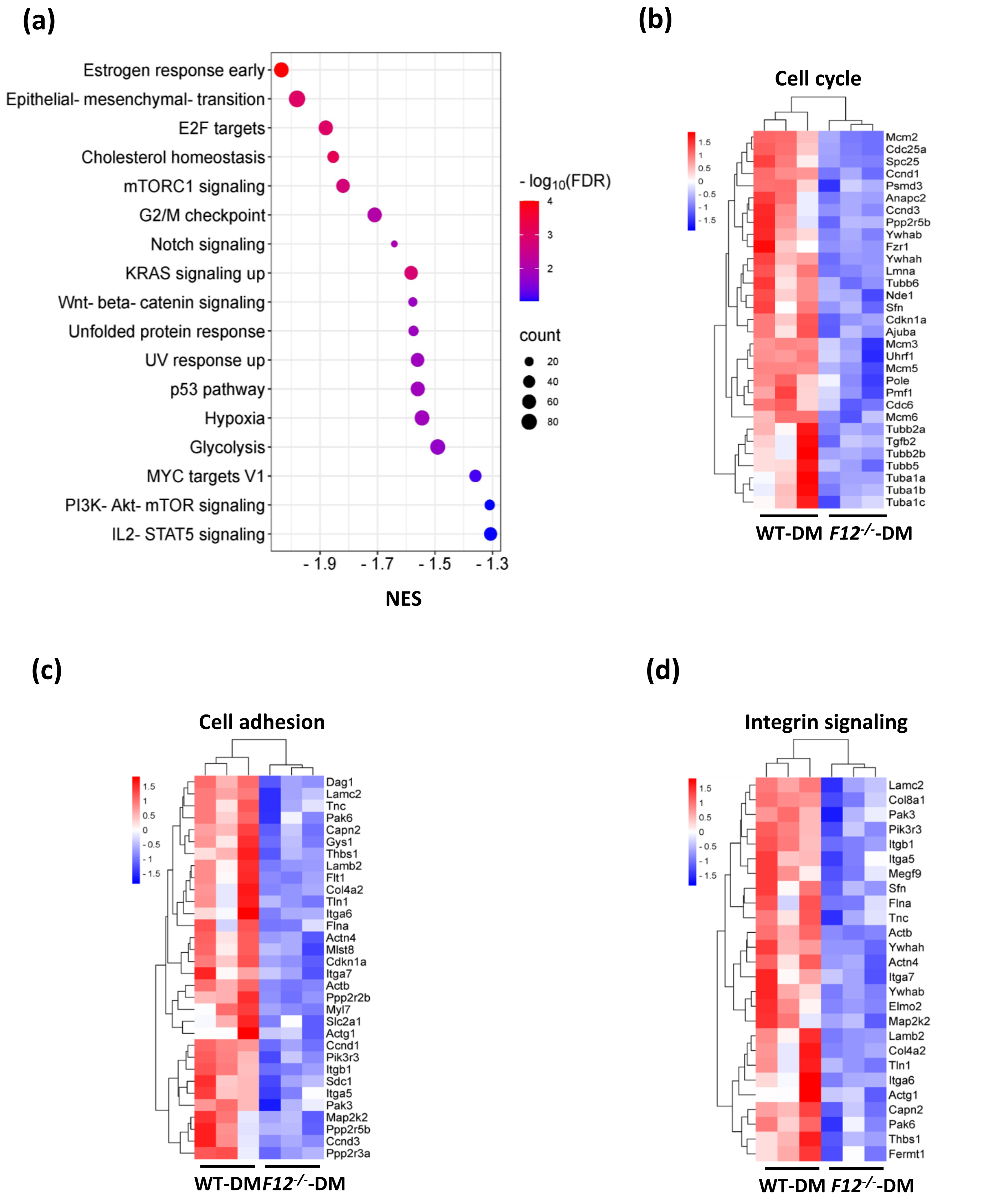

(a)
Estrogen response early
Epithelial- mesenchymal- transition
E2F targets
Cholesterol homeostasis
mTORC1 signaling
G2/M checkpoint
Notch signaling
KRAS signaling up
Wnt- beta- catenin signaling
Unfolded protein response
UV response up
p53 pathway
Hypoxia
Glycolysis
MYC targets V1
PI3K- Akt- mTOR signaling
IL2- STAT5 signaling
- 1.9
- 1.7
- 1.5
- 1.3
NES
- log10(FDR)
4
3
2
count
20
40
60
80
(b)
Cell cycle
1.5
1
0.5
0
- 0.5
- 1
- 1.5
Mcm2
Cdc25a
Spc25
Ccnd1
Psmd3
Anapc2
Ccnd3
Ppp2r5b
Ywhab
Fzr1
Ywhah
Lmna
Tubb6
Nde1
Sfn
Cdkn1a
Ajuba
Mcm3
Uhrf1
Mcm5
Pole
Pmf1
Cdc6
Mcm6
Tubb2a
Tgfb2
Tubb2b
Tubb5
Tuba1a
Tuba1b
Tuba1c
WT-DM
F12-/--DM
(c)
Cell adhesion
1.5
1
0.5
0
- 0.5
- 1
- 1.5
Dag1
Lamc2
Tnc
Pak6
Capn2
Gys1
Thbs1
Lamb2
Flt1
Col4a2
Tln1
Itga6
Flna
Actn4
Mlst8
Cdkn1a
Itga7
Actb
Ppp2r2b
Myl7
Slc2a1
Actg1
Ccnd1
Pik3r3
Itgb1
Sdc1
Itga5
Pak3
Map2k2
Ppp2r5b
Ccnd3
Ppp2r3a
WT-DM
F12-/--DM
(d)
Integrin signaling
1.5
1
0.5
0
- 0.5
- 1
- 1.5
Lamc2
Col8a1
Pak3
Pik3r3
Itgb1
Itga5
Megf9
Sfn
Flna
Tnc
Actb
Ywhah
Actn4
Itga7
Ywhab
Elmo2
Map2k2
Lamb2
Col4a2
Tln1
Itga6
Actg1
Capn2
Pak6
Thbs1
Fermt1
WT-DM
F12-/--DM

Fig. S3 Negative enrichment of DKD-associated pathways in *F12$^{-/-}$* mice

a) Bubble plot showing the negatively enriched pathways in whole kidney transcriptomes of hyperglycemic *F12$^{-/-}$* compared to hyperglycemic WT mice from the Hallmark gene sets obtained by Gene set enrichment analysis (GSEA). The pathways are ordered and represented by normalized enrichment score (NES) and false discovery rate (FDR).

b-d) Heatmaps of the RNA-seq data showing kidney expression changes of cell cycle (b), cell adhesion (c), and integrin signaling (d) related genes in WT-DM and *F12$^{-/-}$* -DM mice. Each column represents data from an individual mouse. Color intensity represents row Z-score.

Fig. S.4

(a)

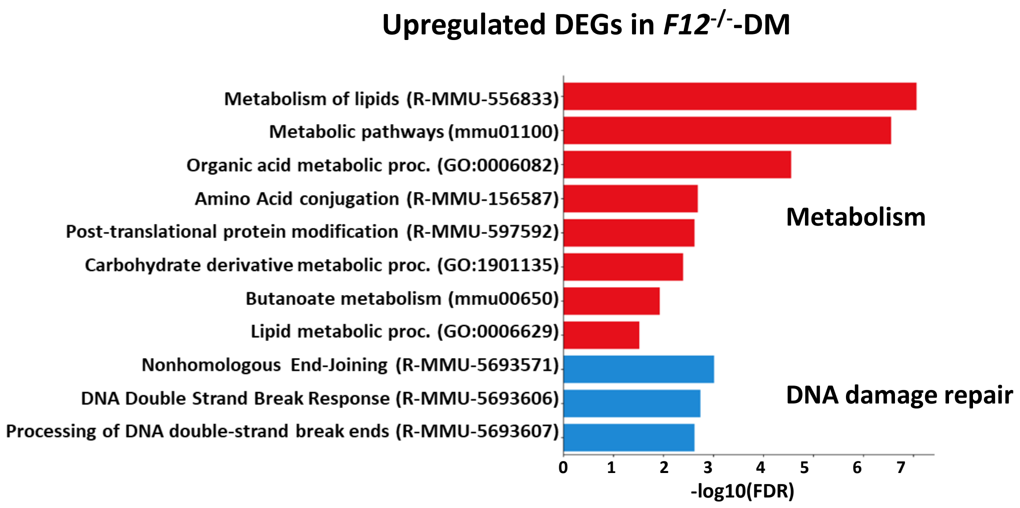
Upregulated DEGs in F12-/--DM
Metabolism of lipids (R-MMU-556833)
Metabolic pathways (mmu01100)
Organic acid metabolic proc. (GO:0006082)
Amino Acid conjugation (R-MMU-156587)
Post-translational protein modification (R-MMU-597592)
Carbohydrate derivative metabolic proc. (GO:1901135)
Butanoate metabolism (mmu00650)
Lipid metabolic proc. (GO:0006629)
Nonhomologous End-Joining (R-MMU-5693571)
DNA Double Strand Break Response (R-MMU-5693606)
Processing of DNA double-strand break ends (R-MMU-5693607)
Metabolism
DNA damage repair
0 1 2 3 4 5 6 7
-log10(FDR)

(b)

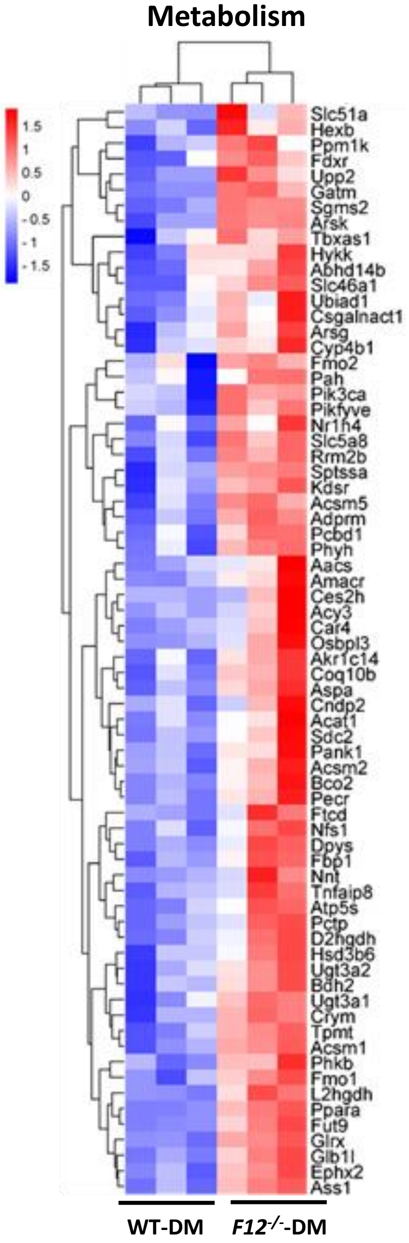
Metabolism
1.5
1
0.5
0
-0.5
-1
-1.5
Slc51a
Hexb
Ppm1k
Fdxr
Upp2
Gatm
Sgms2
Arsk
Tbxas1
Hykk
Abhd14b
Slc46a1
Ubiad1
Csgalnact1
Arsg
Cyp4b1
Fmo2
Pah
Pik3ca
Pikfyve
Nr1h4
Slc5a8
Rrm2b
Sptssa
Kdsr
Acsm5
Adprm
Pcbd1
Phyh
Aacs
Amacr
Ces2h
Acy3
Car4
Osbpl3
Akr1c14
Coq10b
Aspa
Cndp2
Acat1
Sdc2
Pank1
Acsm2
Bco2
Pecr
Ftcd
Nfs1
Dpys
Fbp1
Nnt
Tnfaip8
Atp5s
Pctp
D2hgdh
Hsd3b6
Ugt3a2
Bdh2
Ugt3a1
Crym
Tpmt
Acsm1
Phkb
Fmo1
L2hgdh
Ppara
Fut9
Glrx
Glb1l
Ephx2
Ass1
WT-DM
F12-/--DM

Fig. S4 Differential gene expression in *F12*$^{-/-}$ mice

a) Bar graph representing the top enriched pathways based on the upregulated differentially expressed genes (DEGs) in *F12*$^{-/-}$-DM compared to WT-DM kidneys using KEGG (Kyoto Encyclopedia of Genes and Genomes), Reactome, and GO (Gene Ontology: Biological processes) databases. The pathways were ranked by the false discovery rate (FDR).

b) Heatmap of RNA-seq data showing expression changes of metabolism related genes in WT-DM and *F12*$^{-/-}$-DM mice kidneys. Each column represents data from an individual mouse. Color intensity represents row Z-score.

Fig. S.5

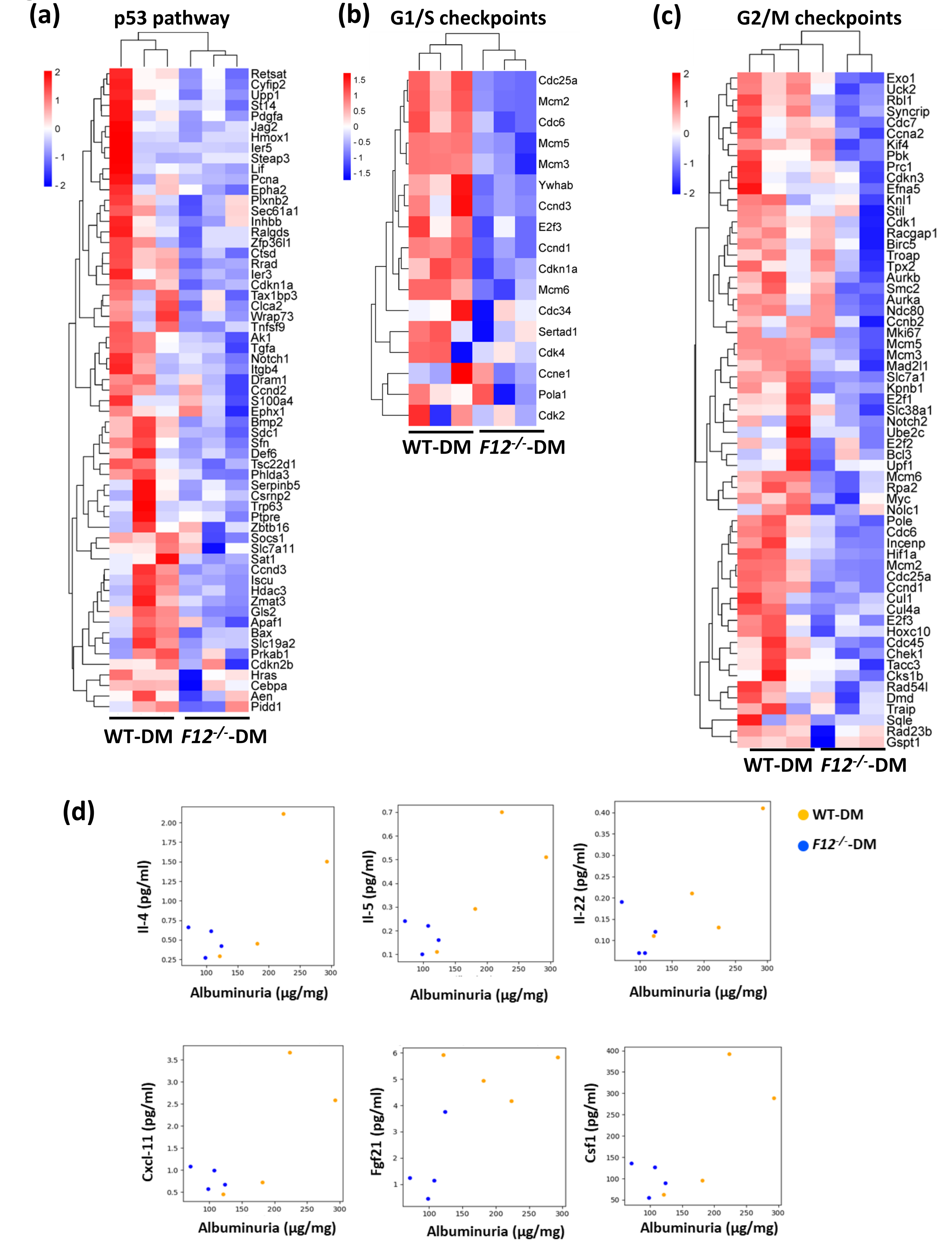

(a)
p53 pathway
Retsat
Cyfip2
Upp1
St14
Pdgfa
Jag2
Hmox1
Ier5
Steap3
Lif
Pcna
Epha2
Plxnb2
Sec61a1
Inhbb
Ralgds
Zfp36l1
Ctsd
Rrad
Ier3
Cdkn1a
Tax1bp3
Clca2
Wrap73
Tnfsf9
Ak1
Tgfa
Notch1
Itgb4
Dram1
Ccnd2
S100a4
Ephx1
Bmp2
Sdc1
Sfn
Def6
Tsc22d1
Phlda3
Serpinb5
Csrnp2
Trp63
Ptpre
Zbtb16
Socs1
Slc7a11
Sat1
Ccnd3
Iscu
Hdac3
Zmat3
Gls2
Apaf1
Bax
Slc19a2
Prkab1
Cdkn2b
Hras
Cebpa
Aen
Pidd1
WT-DM
F12-/--DM
(b)
G1/S checkpoints
Cdc25a
Mcm2
Cdc6
Mcm5
Mcm3
Ywhab
Ccnd3
E2f3
Ccnd1
Cdkn1a
Mcm6
Cdc34
Sertad1
Cdk4
Ccne1
Pola1
Cdk2
WT-DM
F12-/--DM
(c)
G2/M checkpoints
Exo1
Uck2
Rbl1
Syncrip
Cdc7
Ccna2
Kif4
Pbk
Prc1
Cdkn3
Efna5
Knl1
Stil
Cdk1
Racgap1
Birc5
Troap
Tpx2
Aurkb
Smc2
Aurka
Ndc80
Ccnb2
Mki67
Mcm5
Mcm3
Mad2l1
Slc7a1
Kpnb1
E2f1
Slc38a1
Notch2
Ube2c
E2f2
Bcl3
Upf1
Mcm6
Rpa2
Myc
Nolc1
Pole
Cdc6
Incenp
Hif1a
Mcm2
Cdc25a
Ccnd1
Cul1
Cul4a
E2f3
Hoxc10
Cdc45
Chek1
Tacc3
Cks1b
Rad54l
Dmd
Traip
Sqle
Rad23b
Gspt1
WT-DM
F12-/--DM
(d)
WT-DM
F12-/--DM
Il-4 (pg/ml)
Il-5 (pg/ml)
Il-22 (pg/ml)
Cxcl-11 (pg/ml)
Fgf21 (pg/ml)
Csf1 (pg/ml)
Albuminuria (µg/mg)

Fig. S5 Cell cycle arrest genes are negatively enriched in *F12*$^{-/-}$ mice

a-c) Heatmap of the RNA-seq data showing expression changes of p53 pathway (a), G1/S cell cycle checkpoints (b), and G2/M cell cycle checkpoints (c) related genes in WT-DM and *F12*$^{-/-}$-DM mice kidneys. Each column represents data from an individual mouse. Color intensity represents row Z-score.

d) Scatter plots representing the expression of selected SASP-related factors in kidney lysates in relation to albuminuria levels in hyperglycemic WT and *F12*$^{-/-}$ mice. The SASP factors were detected using Olink technology.

Fig. S.6

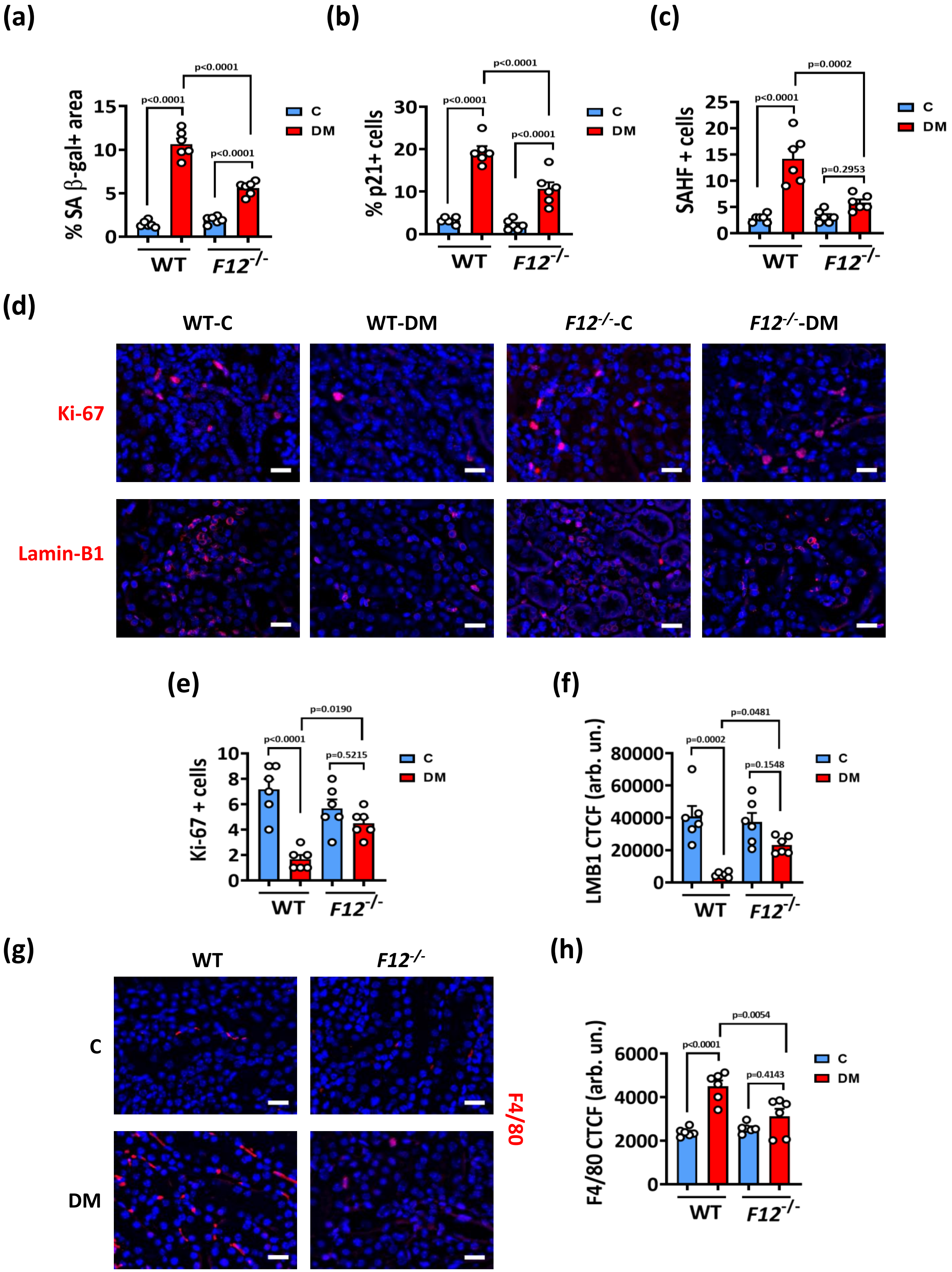

(a)
% SA β-gal+ area
p<0.0001
p<0.0001
p<0.0001
C
DM
WT
F12-/-
(b)
% p21+ cells
p<0.0001
p<0.0001
p<0.0001
(c)
SAHF + cells
p=0.0002
p<0.0001
p=0.2953
(d)
WT-C
WT-DM
F12-/--C
F12-/--DM
Ki-67
Lamin-B1
(e)
Ki-67 + cells
p=0.0190
p<0.0001
p=0.5215
(f)
LMB1 CTCF (arb. un.)
p=0.0481
p=0.0002
p=0.1548
(g)
WT
F12-/-
C
DM
F4/80
(h)
F4/80 CTCF (arb. un.)
p=0.0054
p<0.0001
p=0.4143

Fig. S6 *F12*$^{-/-}$ mice kidneys are less susceptible to DKD-associated senescence

a-c) Dot plots summarizing quantification of senescence associated β-galactosidase (SA-β-gal; a), p21 (b), and senescence-associated heterochromatin foci of tri-methyl-histone H3 (Lys9) (SAHF; c) in normoglycemic control (C) and hyperglycemic (DM) WT and *F12*$^{-/-}$ mice. Dot plots reflecting mean±SEM of 6 mice per group; two-way ANOVA with Tukeys's multiple comparison test.

d-f) Exemplary histological images (d) and dot plots summarizing results (e,f) of the proliferation marker Ki-67 (top panel) and the nuclear membrane marker lamin-B1 (bottom panel) in experimental groups (as described in a). Ki-67 and lamin-B1 are immunofluorescently detected, red; DAPI nuclear counterstain, blue. Scale bars represent 20 µm. Dot plots reflecting mean±SEM of 6 mice per group; two-way ANOVA with Tukeys's multiple comparison test. CTCF: corrected total cell fluorescence. Arb. un.: arbitrary units..

g,h) Exemplary histological images (g) and dot plot summarizing results (h) of the macrophage marker F4/80 in experimental groups (as described in a). F4/80 is immunofluorescently detected, red; DAPI nuclear counterstain, blue. Scale bars represent 20 µm. Dot plot reflecting mean±SEM of 6 mice per group; two-way ANOVA with Tukeys's multiple comparison test. CTCF: corrected total cell fluorescence. Arb. Un.: arbitrary units.

Fig. S.7

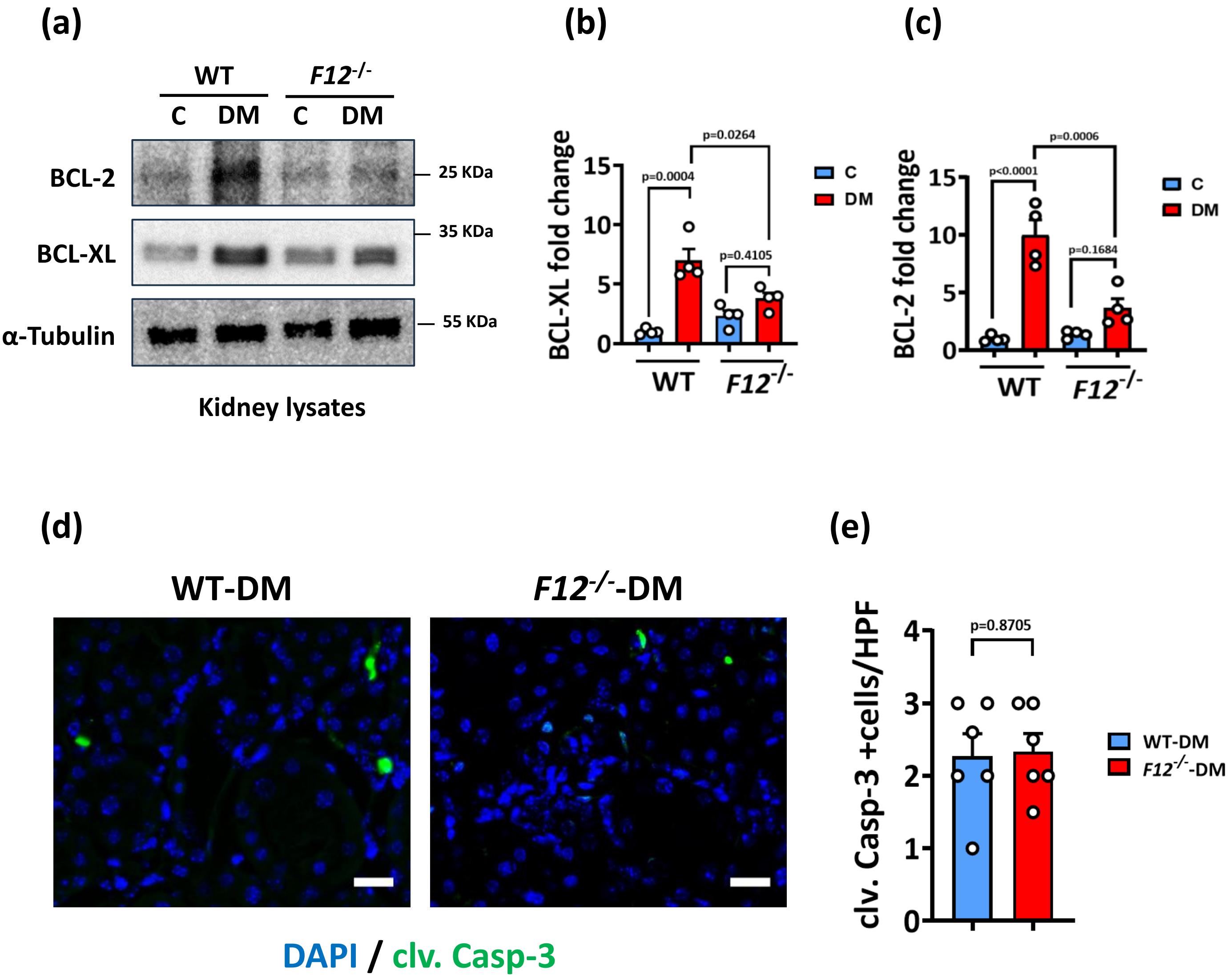

(a)
WT
F12-/-
C
DM
C
DM
BCL-2
25 KDa
BCL-XL
35 KDa
α-Tubulin
55 KDa
Kidney lysates
(b)
BCL-XL fold change
p=0.0264
p=0.0004
p=0.4105
C
DM
WT
F12-/-
(c)
BCL-2 fold change
p=0.0006
p<0.0001
p=0.1684
C
DM
WT
F12-/-
(d)
WT-DM
F12-/--DM
DAPI / clv. Casp-3
(e)
clv. Casp-3 +cells/HPF
p=0.8705
WT-DM
F12-/--DM

Fig. S7 FXII upregulation in diabetic kidneys is not associated with apoptosis

(a-c) Representative immunoblots (a, loading control: α-Tubulin) and dot plots summarizing results for BCL-XL (b) and BCL-2 (c) expression in kidney lysates of normoglycemic controls (C) and hyperglycemic (DM) wild type (WT) and $F12^{-/-}$ mice. Dot plot reflecting mean±SEM of 4 mice per group; two-way ANOVA with Tukeys's multiple comparison test.

(d,e) Exemplary histological images (d) and dot plot summarizing results (e) of the cleaved Caspase-3 (clv. Casp-3) positive cells in hyperglycemic (DM) wild type (WT) and $F12^{-/-}$ mice. clv. Casp-3 is immunofluorescently detected, green; DAPI nuclear counterstain, blue. Scale bars represent 20 µm. Dot plot reflecting mean±SEM of 6 mice per group; two-tailed unpaired student's t-test.

Fig. S.8

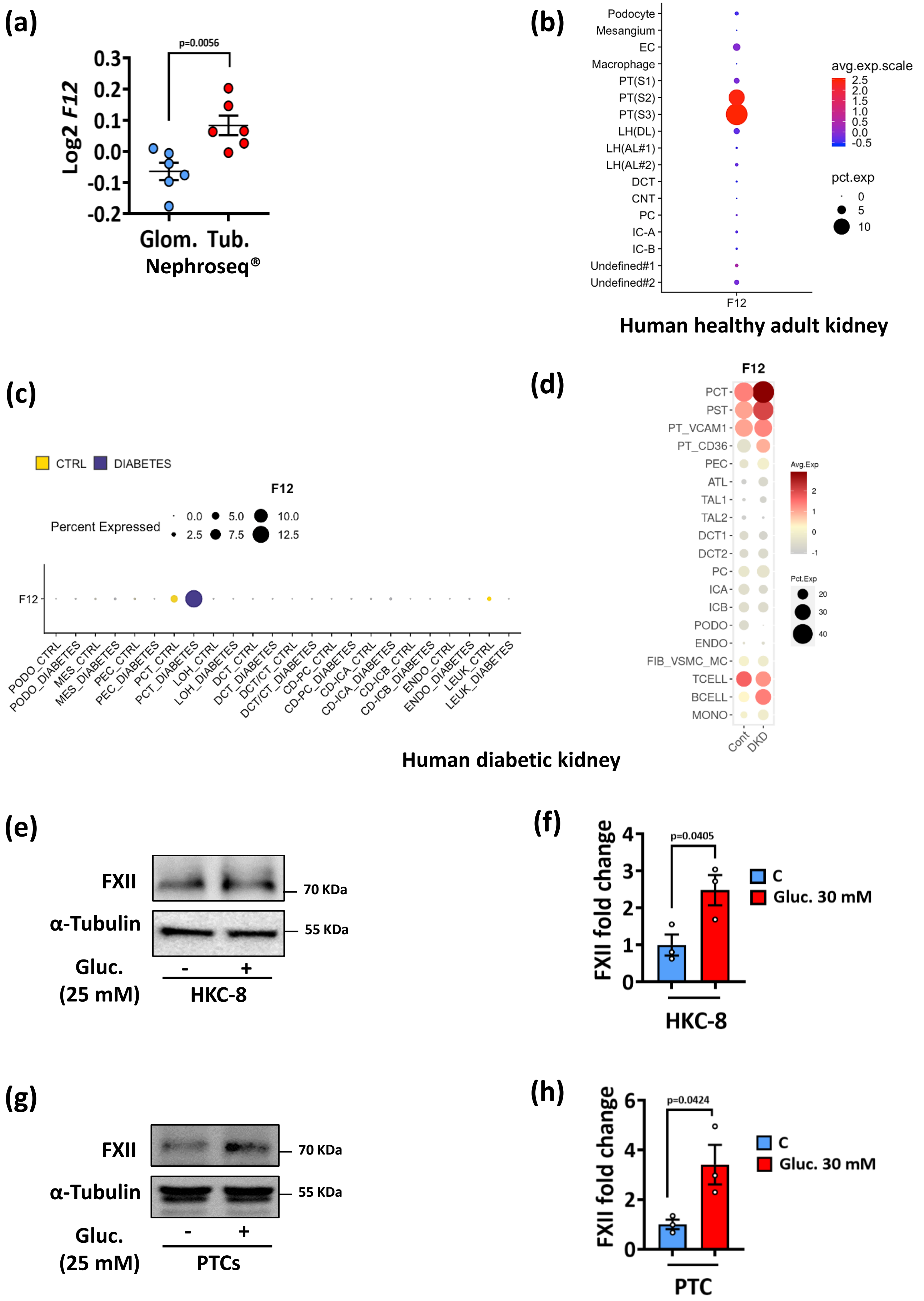
(a)
p=0.0056
Log2 F12
0.3
0.2
0.1
0.0
-0.1
-0.2
Glom. Tub.
Nephroseq®
(b)
Podocyte
Mesangium
EC
Macrophage
PT(S1)
PT(S2)
PT(S3)
LH(DL)
LH(AL#1)
LH(AL#2)
DCT
CNT
PC
IC-A
IC-B
Undefined#1
Undefined#2
F12
avg.exp.scale
2.5
2.0
1.5
1.0
0.5
0.0
-0.5
pct.exp
0
5
10
Human healthy adult kidney
(c)
CTRL DIABETES
F12
Percent Expressed
0.0 5.0 10.0
2.5 7.5 12.5
F12
PODO_CTRL
PODO_DIABETES
MES_CTRL
MES_DIABETES
PEC_CTRL
PEC_DIABETES
PCT_CTRL
PCT_DIABETES
LOH_CTRL
LOH_DIABETES
DCT_CTRL
DCT_DIABETES
DCT/CT_CTRL
DCT/CT_DIABETES
CD-PC_CTRL
CD-PC_DIABETES
CD-ICA_CTRL
CD-ICA_DIABETES
CD-ICB_CTRL
CD-ICB_DIABETES
ENDO_CTRL
ENDO_DIABETES
LEUK_CTRL
LEUK_DIABETES
(d)
F12
PCT
PST
PT_VCAM1
PT_CD36
PEC
ATL
TAL1
TAL2
DCT1
DCT2
PC
ICA
ICB
PODO
ENDO
FIB_VSMC_MC
TCELL
BCELL
MONO
Cont
DKD
Avg.Exp
2
1
0
-1
Pct.Exp
20
30
40
Human diabetic kidney
(e)
FXII
α-Tubulin
70 KDa
55 KDa
Gluc.
(25 mM)
-
+
HKC-8
(f)
p=0.0405
FXII fold change
4
3
2
1
0
C
Gluc. 30 mM
HKC-8
(g)
FXII
α-Tubulin
70 KDa
55 KDa
Gluc.
(25 mM)
-
+
PTCs
(h)
p=0.0424
FXII fold change
6
4
2
0
C
Gluc. 30 mM
PTC

Fig. S8 FXII is predominantly expressed by human and murine kidney tubular cells

a) Dot plot summarizing *F12* expression in the glomerular (Glom.) and tubulointerstitial (Tub.) compartments (Nephroseq® database). Dot plot reflecting mean±SEM; two-tailed unpaired student’s t-test.

b-d) Dot plots reflecting differential cellular *F12* expression in different single cell sequencing transcriptomic datasets in healthy adult human kidneys (b) and comparing diabetic kidney disease patients to non-diabetic controls (c,d). Data are obtained from the Kidney Interactive Transcriptomics database (KIT).

e,f) Representative immunoblots (e, loading control: α-Tubulin) and dot plot summarizing results (f) for FXII expression in HKC-8 cells exposed to high glucose (25 mM) for 24 h compared to control cells (5 mM glucose). Dot plot reflecting mean±SEM of 3 independent experiments; two-tailed unpaired student’s t-test.

g,h) Representative immunoblots (g, loading control: α-Tubulin) and dot plot summarizing results (h) for FXII expression in mouse primary tubular cells (PTCs) exposed to high glucose (25 mM) for 24 h compared to control cells (5 mM glucose). Dot plot reflecting mean±SEM of 3 independent experiments; two-tailed unpaired student’s t-test.

Fig. S.9

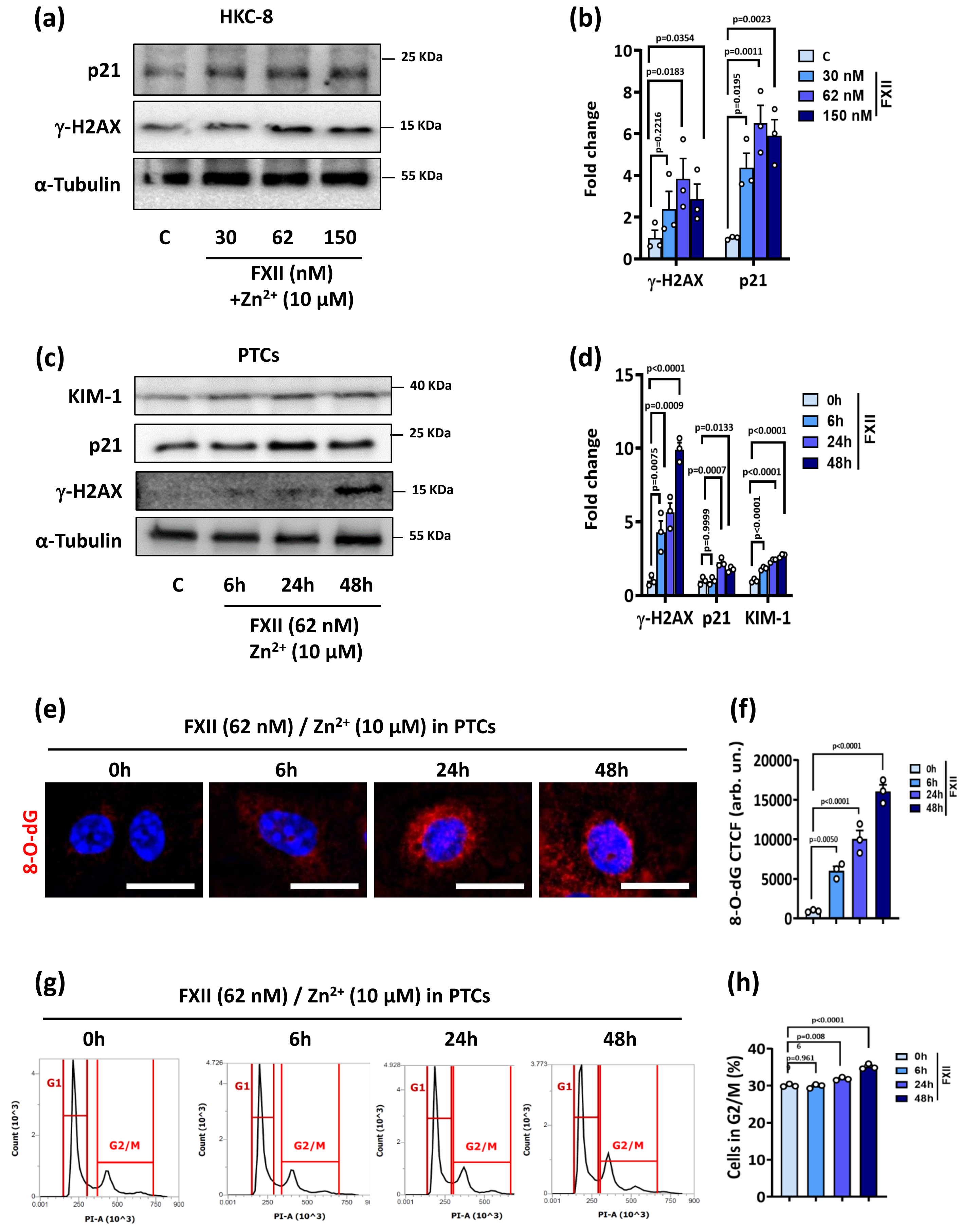
(a)
HKC-8
p21
γ-H2AX
α-Tubulin
25 KDa
15 KDa
55 KDa
C
30
62
150
FXII (nM)
+Zn2+ (10 μM)
(b)
Fold change
γ-H2AX
p21
C
30 nM
62 nM
150 nM
FXII
p=0.0354
p=0.0183
p=0.2216
p=0.0023
p=0.0011
p=0.0195
(c)
PTCs
KIM-1
p21
γ-H2AX
α-Tubulin
40 KDa
25 KDa
15 KDa
55 KDa
C
6h
24h
48h
FXII (62 nM)
Zn2+ (10 μM)
(d)
Fold change
γ-H2AX
p21
KIM-1
0h
6h
24h
48h
FXII
p<0.0001
p=0.0009
p=0.0075
p=0.0133
p=0.0007
p=0.9999
p<0.0001
p<0.0001
p<0.0001
(e)
FXII (62 nM) / Zn2+ (10 μM) in PTCs
0h
6h
24h
48h
8-O-dG
(f)
8-O-dG CTCF (arb. un.)
p<0.0001
p<0.0001
p=0.0050
0h
6h
24h
48h
FXII
(g)
FXII (62 nM) / Zn2+ (10 μM) in PTCs
0h
6h
24h
48h
G1
G2/M
Count (10^3)
PI-A (10^3)
(h)
Cells in G2/M (%)
p<0.0001
p=0.008
p=0.961
0h
6h
24h
48h
FXII

Fig. S9 FXII induces senescence in human and murine kidney tubular cells

a,b) Representative immunoblots (a, loading control: α-Tubulin) and dot plots summarizing results (b) for γ-H2AX and p21 expression in HKC-8 cells exposed to purified human FXII (30, 62, or 150 nM) in the presence of Zn^{2+} (10 µM) for 24 h. Dot plots reflecting mean±SEM of 3 independent experiments; one-way ANOVA with Tukeys's multiple comparison test.

c,d) Representative immunoblots (c, loading control: α-Tubulin) and dot plots summarizing results (d) for γ-H2AX, p21, and KIM-1 expression in mouse primary tubular cells (PTCs) exposed to recombinant mouse FXII (62 nM) in the presence of Zn^{2+} (10 µM) for 6, 24, and 48 h. Dot plots reflecting mean±SEM of 3 independent experiments; one-way ANOVA with Tukeys's multiple comparison test.

e,f) Exemplary images (e) and dot plots summarizing results (f) of 8-hydroxy-2'-deoxyguanosine (8-O-dG) staining in experimental groups (as described in c). 8-O-dG is immunofluorescently detected, red; DAPI nuclear counterstain, blue. Scale bars represent 20 µm. Dot plots reflecting mean±SEM of 3 independent experiments; one-way ANOVA with Tukeys's multiple comparison test. CTCF: corrected total cell fluorescence. Arb. un.: arbitrary units.

g) Representative histograms summarizing the distribution of cells in different cell cycle phases determined by flow cytometry as counts in mouse primary tubular cells (PTCs) exposed to recombinant mouse FXII (62 nM) in the presence of Zn^{2+} (10 µM) for 6, 24, and 48 h and stained with promidium iodide compared to control cells.

h) Dot plot summarizing the percentage of G2/M phase cells of the cell cycle analysis using flow cytometry of promidium iodide staining in PTCs (as described in g). Dot plots reflecting mean±SEM of 3 independent experiments; one-way ANOVA with Tukeys's multiple comparison test.

Fig. S.10

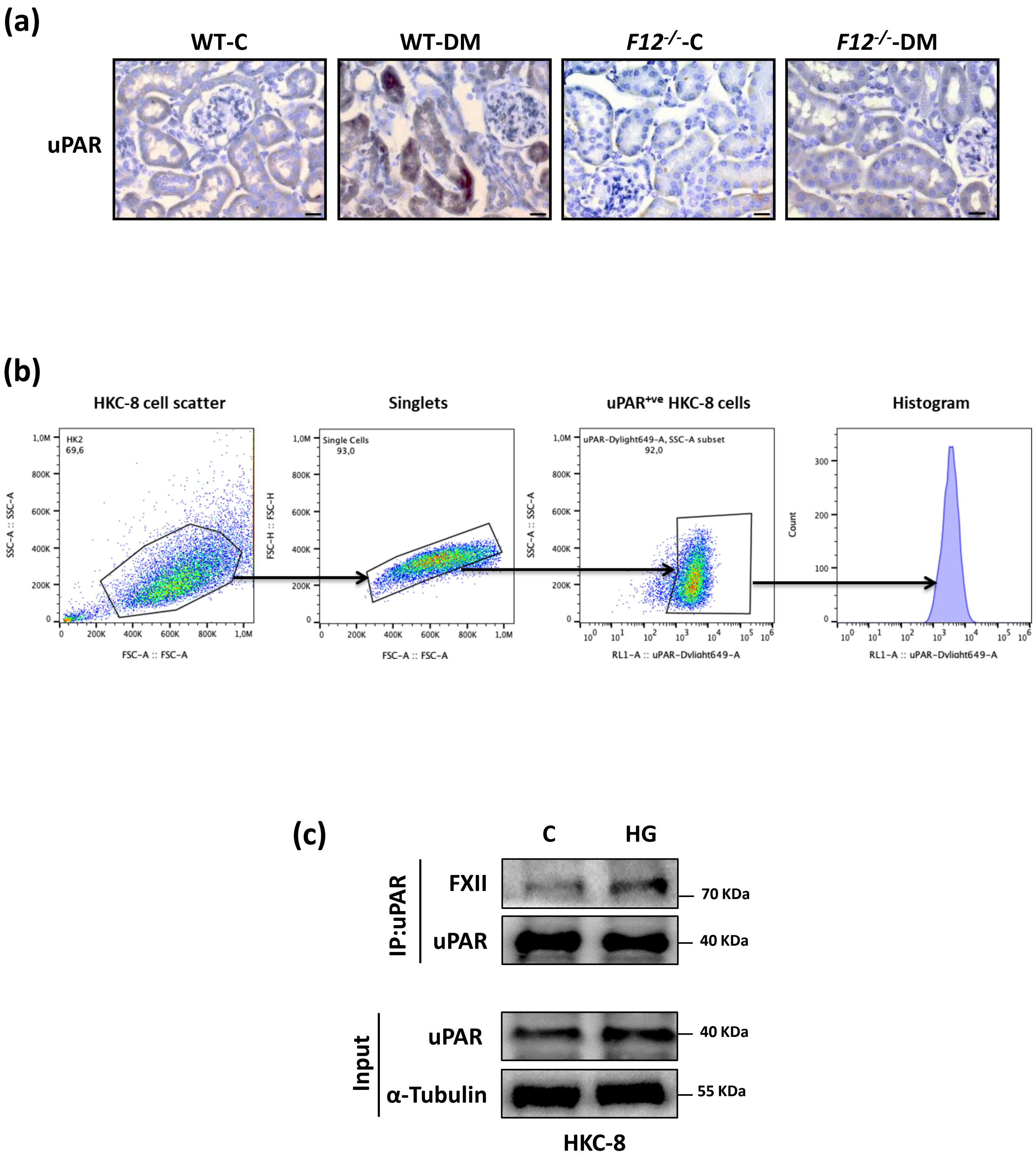

(a)
WT-C
WT-DM
F12-/--C
F12-/--DM
uPAR
(b)
HKC-8 cell scatter
Singlets
uPAR+ve HKC-8 cells
Histogram
HK2
69,6
Single Cells
93,0
uPAR-Dylight649-A, SSC-A subset
92,0
SSC-A :: SSC-A
FSC-H :: FSC-H
Count
FSC-A :: FSC-A
RL1-A :: uPAR-Dylight649-A
1,0M
800K
600K
400K
200K
0
300
200
100
(c)
C
HG
IP:uPAR
FXII
uPAR
70 KDa
40 KDa
Input
uPAR
α-Tubulin
40 KDa
55 KDa
HKC-8

Fig. S10 FXII interacts with uPAR on kidney tubular cells

a) Exemplary histological images of mouse kidney sections stained for uPAR in normoglycemic control (C) and hyperglycemic (DM) WT and *F12*$^{-/-}$ mice. FXII is detected by HRP-DAB reaction (brown); hematoxylin nuclear counter stain (blue). Scale bars represent 20 µm. Images represent 6 mice per group.

b) Gating strategy for uPAR surface staining of HKC-8 cells by flow cytometry.

c) Representative immunoblots for FXII and uPAR from uPAR coimmunoprecipitation (IP, top) and immunoblots for uPAR and α-Tubulin from input (input, bottom) in HKC-8 cells exposed to normal (5 mM, C) or high glucose (25 mM, HG) for 24 h. Input serves as a loading control. Immunoblots representing 3 independent experiments.

Fig. S.11

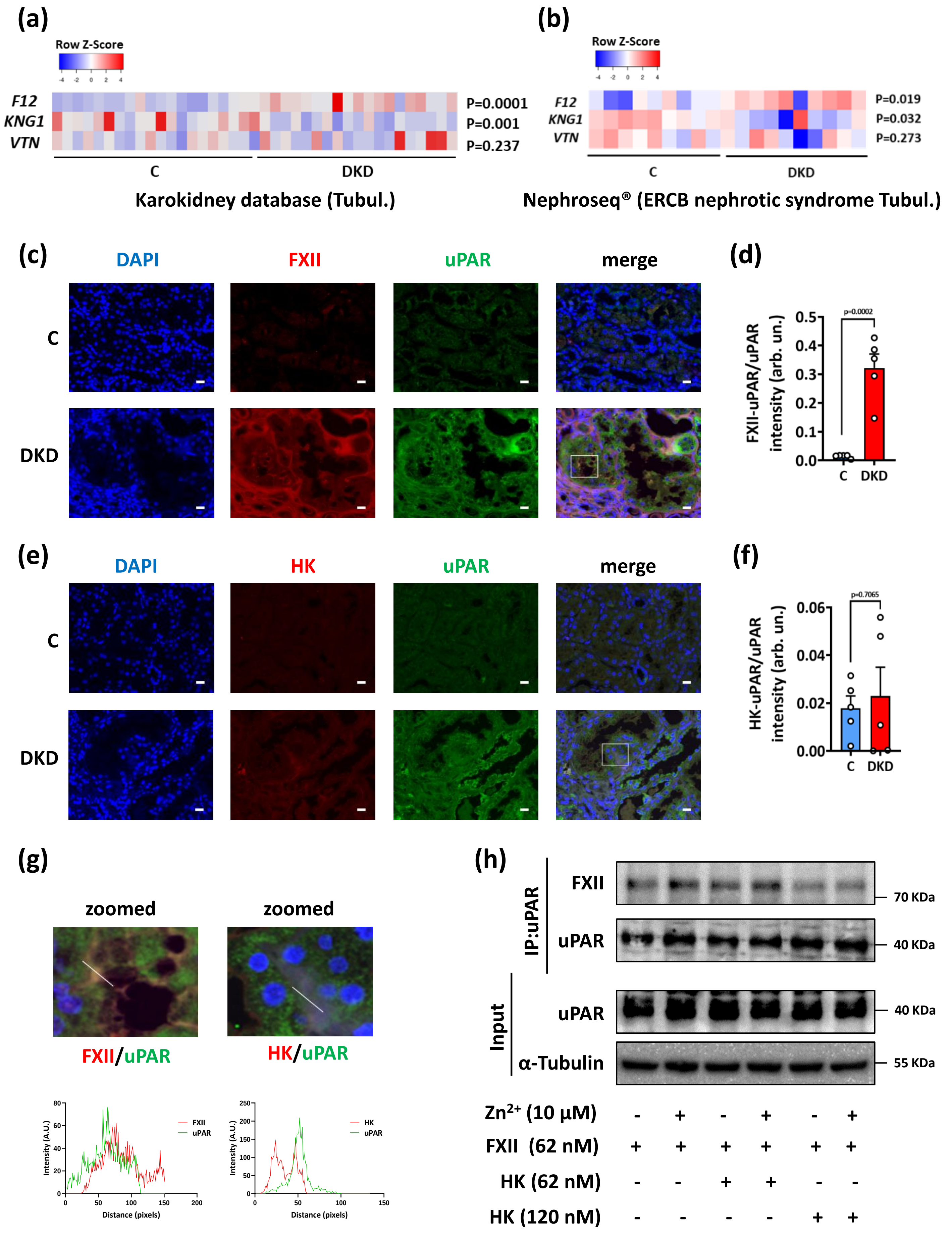

(a)
Row Z-Score
F12
KNG1
VTN
P=0.0001
P=0.001
P=0.237
C
DKD
Karokidney database (Tubul.)
(b)
Row Z-Score
F12
KNG1
VTN
P=0.019
P=0.032
P=0.273
C
DKD
Nephroseq® (ERCB nephrotic syndrome Tubul.)
(c)
DAPI
FXII
uPAR
merge
C
DKD
(d)
p=0.0002
FXII-uPAR/uPAR intensity (arb. un.)
C
DKD
(e)
DAPI
HK
uPAR
merge
C
DKD
(f)
p=0.7065
HK-uPAR/uPAR intensity (arb. un.)
C
DKD
(g)
zoomed
zoomed
FXII/uPAR
HK/uPAR
Intensity (A.U.)
Distance (pixels)
FXII
uPAR
HK
uPAR
(h)
IP:uPAR
FXII
uPAR
70 KDa
40 KDa
Input
uPAR
α-Tubulin
40 KDa
55 KDa
Zn2+ (10 μM)
FXII (62 nM)
HK (62 nM)
HK (120 nM)

Fig. S11 HK does not interfere with FXII-uPAR signaling in DKD

a,b) Heatmaps summarizing *F12*, *KNG1*, and *VTN* expression in the tubulointerstitial compartment comparing diabetic kidney disease patients (DKD) to non-diabetic control healthy donors (C) in Karokidney database (a) and ERCB nephrotic syndrome of the Nephroseq® database (b). Tow-tailed unpaired student's t-test. Color intensity represents row Z-score.

(c-f) Representative histological images of FXII (red) and uPAR (green; c) or HK (red) and uPAR (green; e) in human kidney sections of nondiabetic controls (C) or diabetic patients with DKD (DKD); DAPI nuclear counterstain, blue. Scale bars represent 20 µm, and dot plots summarizing the colocalization intensity to the uPAR intensity in both conditions (d,f). Dot plots reflecting mean±SEM of 5 sections per group; two-tailed unpaired student's t-test.

(g) Magnified areas of the stainings (represented by the white squares in c and e) and histograms showing the intensities of the of FXII (red)-uPAR (green) or HK (red)-uPAR (green) across the drawn solid lines represented in the magnified images.

(h) Representative immunoblots for FXII and uPAR from uPAR coimmunoprecipitation (IP, top) and immunoblots for uPAR, and α-Tubulin from the input (input, bottom) of HKC-8 cells exposed to purified human FXII (62 nM; in the presence and absence of zinc, 10 µM), in the presence of equimolar (62 nM) or excess molar (120 nM) concentrations of HK. Input serves as a loading control. Immunoblots represent 3 independent experiments.

Fig. S.12

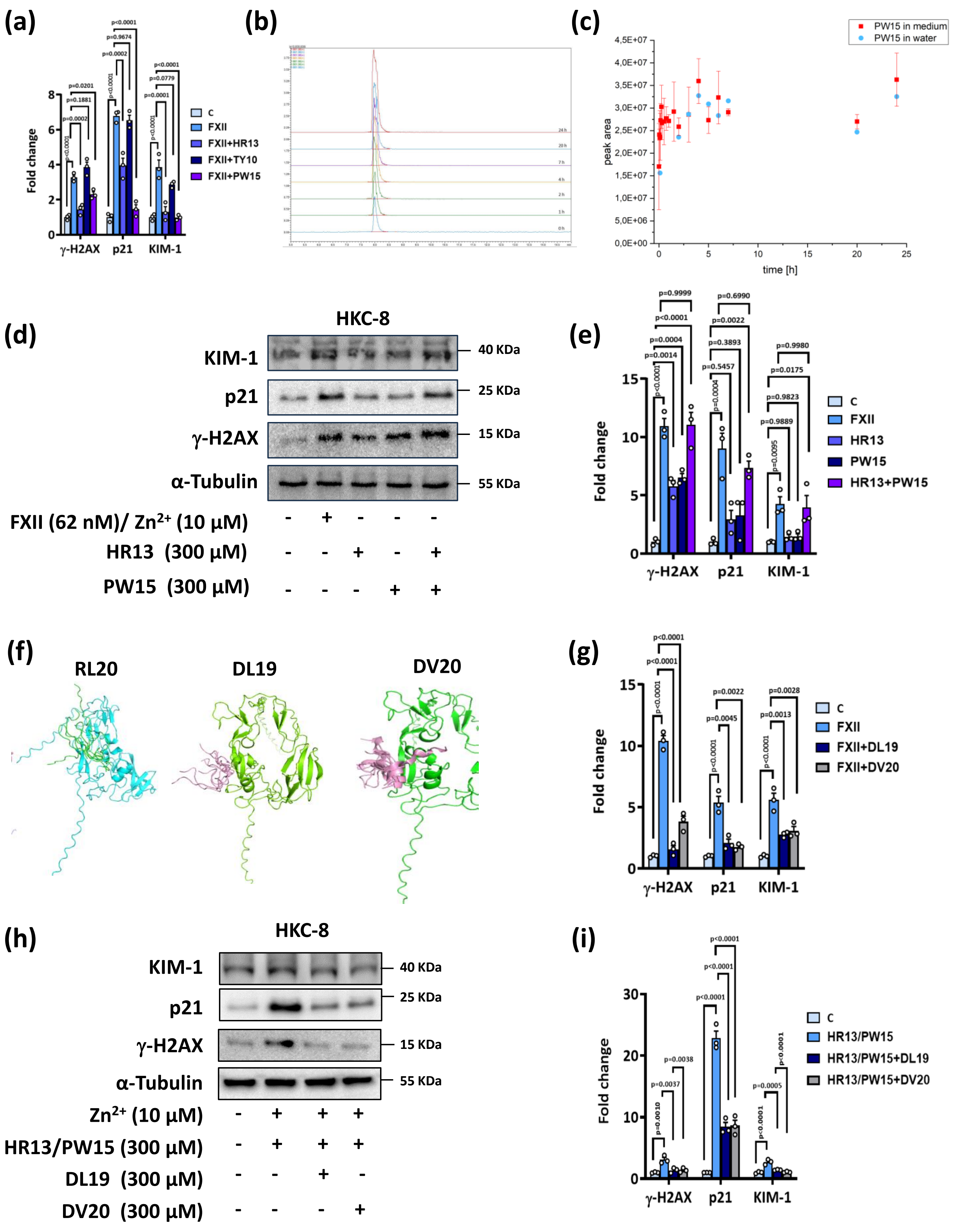
(a)
p<0.0001
p=0.9674
p=0.0002
p<0.0001
p=0.0779
p=0.0001
p=0.0201
p=0.1881
p=0.0002
p<0.0001
p<0.0001
p<0.0001
Fold change
γ-H2AX
p21
KIM-1
C
FXII
FXII+HR13
FXII+TY10
FXII+PW15
(b)
24 h
20 h
7 h
4 h
2 h
1 h
0 h
(c)
PW15 in medium
PW15 in water
peak area
time [h]
(d)
HKC-8
KIM-1
p21
γ-H2AX
α-Tubulin
40 KDa
25 KDa
15 KDa
55 KDa
FXII (62 nM)/ Zn2+ (10 µM)
HR13 (300 µM)
PW15 (300 µM)
(e)
p=0.9999
p<0.0001
p=0.0004
p=0.0014
p<0.0001
p=0.6990
p=0.0022
p=0.3893
p=0.5457
p=0.0004
p=0.9980
p=0.0175
p=0.9823
p=0.9889
p=0.0095
Fold change
γ-H2AX
p21
KIM-1
C
FXII
HR13
PW15
HR13+PW15
(f)
RL20
DL19
DV20
(g)
p<0.0001
p<0.0001
p<0.0001
p=0.0022
p=0.0045
p<0.0001
p=0.0028
p=0.0013
p<0.0001
Fold change
γ-H2AX
p21
KIM-1
C
FXII
FXII+DL19
FXII+DV20
(h)
HKC-8
KIM-1
p21
γ-H2AX
α-Tubulin
40 KDa
25 KDa
15 KDa
55 KDa
Zn2+ (10 µM)
HR13/PW15 (300 µM)
DL19 (300 µM)
DV20 (300 µM)
(i)
p<0.0001
p<0.0001
p<0.0001
p=0.0038
p=0.0037
p=0.0010
p=0.0005
p<0.0001
p<0.0001
Fold change
γ-H2AX
p21
KIM-1
C
HR13/PW15
HR13/PW15+DL19
HR13/PW15+DV20

Fig. S12 FXII requires FN2 and kringle domains to interact with uPAR on tubular cells

a) Dot plots summarizing quantification for γ-H2AX, p21, and KIM-1 expression in HKC-8 cells exposed to purified human FXII (62nM) in the presence of Zn^{2+} (10 µM) for 24 h with and without pretreatment with FXII sequential peptides (HR13, TY10 and PW15; 300µM) for 1 h compared to control untreated cells (C). Dot plots reflecting mean±SEM of 3 independent experiments; one-way ANOVA with Tukeys's multiple comparison test.

b,c) Mass chromatogram of the SIM (selected ion monitoring) of PW15 (661.9 m/z) at different time points as detected by LC/MS (b) and a graph representing the peak areas calculated based on UV detection of PW15 at 215 nm at different time points in water or in HKC-8 medium (c).

d,e) Representative immunoblots (a, loading control: α-Tubulin) and dot plot summarizing results (c) for γ-H2AX, p21, and KIM-1 expression in HKC-8 cells exposed to peptides HR13, PW15 or a combination of both (300 µM) in the presence of Zn^{2+} (10 µM) for 24 h compared to control non-treated cells. Dot plots reflecting mean±SEM of 3 independent experiments; one-way ANOVA with Tukeys's multiple comparison test.

f) Representative images of the AlphaFold2_multimer_v3 prediction of uPAR-based peptides binding to the heavy chain of FXII.

g) Dot plots summarizing quantification for γ-H2AX, p21, and KIM-1 expression in HKC-8 cells exposed to purified human FXII (62nM) in the presence of Zn^{2+} (10 µM) for 24 h with and without pretreatment with FXII uPAR-based peptides (RL20, DL19 and DV20; 300µM) for 1 h compared to control untreated cells (C). Dot plots reflecting mean±SEM of 3 independent experiments; one-way ANOVA with Tukeys's multiple comparison test.

h,i) Representative immunoblots (h, loading control: α-Tubulin) and dot plot summarizing results (i) for γ-H2AX, p21, and KIM-1 expression in HKC-8 cells exposed to a combination of peptides HR13 and PW15 (300 µM) in the presence of Zn^{2+} (10 µM) for 24 h with and without pretreatment with FXII uPAR-based peptides DL19 and DV20 (300µM) for 1 h compared to control untreated cells. Dot plots reflecting mean±SEM of 3 independent experiments; one-way ANOVA with Tukeys's multiple comparison test.

Fig. S.13

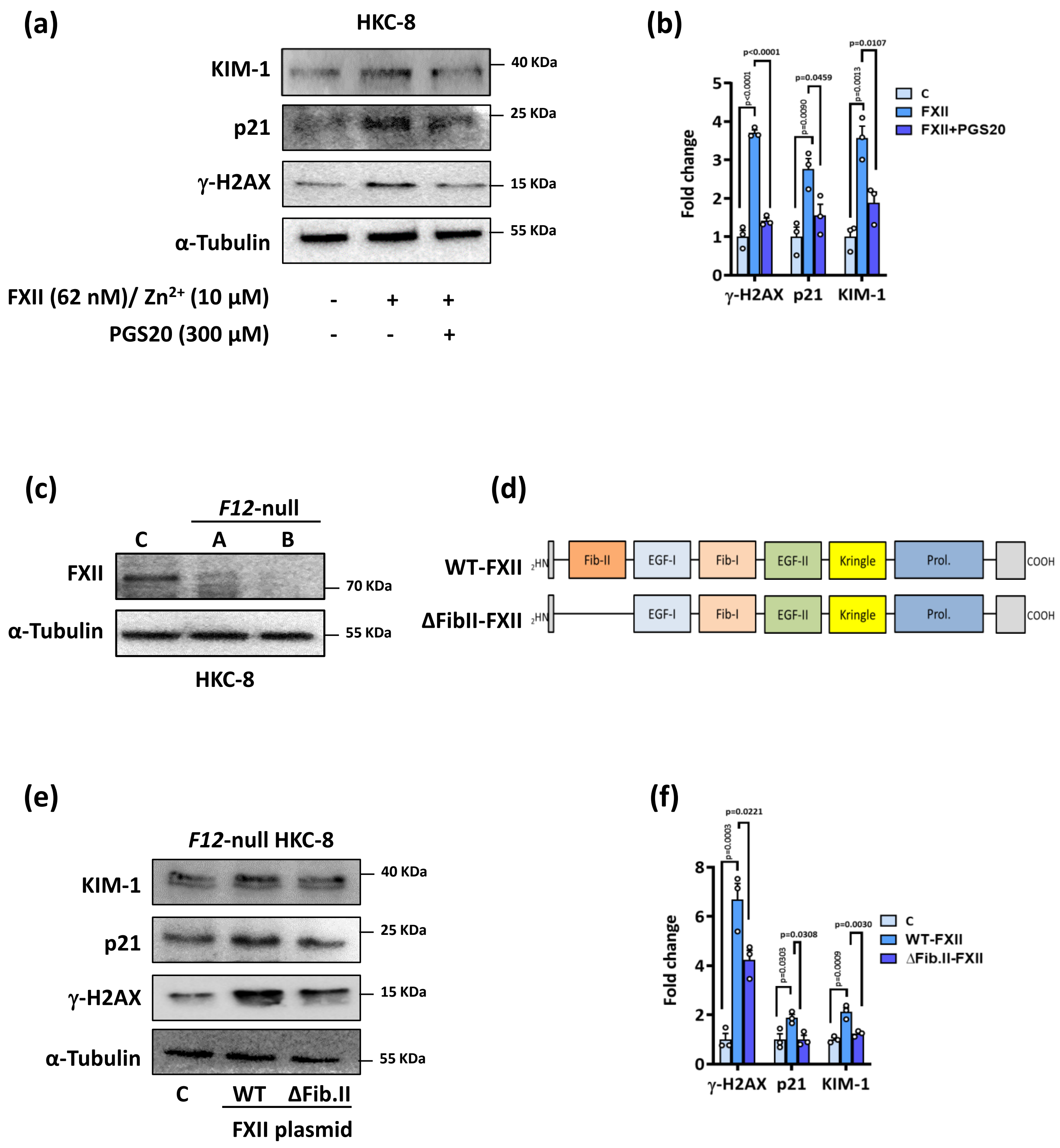
(a)
HKC-8
KIM-1
p21
γ-H2AX
α-Tubulin
40 KDa
25 KDa
15 KDa
55 KDa
FXII (62 nM)/ Zn2+ (10 µM) - + +
PGS20 (300 µM) - - +
(b)
p<0.0001
p=0.0459
p=0.0107
p<0.0001
p=0.0090
p=0.0013
Fold change
γ-H2AX p21 KIM-1
C
FXII
FXII+PGS20
(c)
F12-null
C A B
FXII
α-Tubulin
70 KDa
55 KDa
HKC-8
(d)
WT-FXII
ΔFibII-FXII
Fib-II
EGF-I
Fib-I
EGF-II
Kringle
Prol.
COOH
(e)
F12-null HKC-8
KIM-1
p21
γ-H2AX
α-Tubulin
40 KDa
25 KDa
15 KDa
55 KDa
C WT ΔFib.II
FXII plasmid
(f)
p=0.0221
p=0.0003
p=0.0308
p=0.0030
p=0.0303
p=0.0009
Fold change
γ-H2AX p21 KIM-1
C
WT-FXII
ΔFib.II-FXII

Fig. S13 The relevance of FN2 domain of FXII and uPAR domain 2 for the interaction

a,b) Representative immunoblots (loading control: α-Tubulin, a) and dot plot summarizing results (b) for γ-H2AX, p21, and KIM-1 expression in HKC-8 cells exposed to purified human FXII (62nM) in the presence of Zn^{2+} (10 μM) for 24 h with and without pretreatment with the human uPAR domain 2 blocking peptide; PGS20 (300 μM) for 30 min. Dot plots reflecting mean±SEM of 3 independent experiments; one-way ANOVA with Tukeys's multiple comparison test.

c) Representative immunoblots (loading control: α-Tubulin) for FXII expression in HKC-8 cells transfected with human FXII CRISPR/Cas9 knockout plasmid showing 2 different clones (clone A and B; *F12*-null cells) compared to control non-transfected cells (C).

d) Scheme representing the domains of the zymogen human FXII (WT-FXII) and the mutant lacking the fibronectin type II domain (ΔFib-II-FXII).

e,f) Representative immunoblots (loading control: α-Tubulin, e) and dot plot summarizing results (f) for γ-H2AX, p21, and KIM-1 expression in *F12*-null HKC-8 cells transfected with WT-FXII or ΔFib-II-FXII compared to empty-vector transfected cells (C). Dot plots reflecting mean±SEM of 3 independent experiments; one-way ANOVA with Tukeys's multiple comparison test.

Fig. S.14

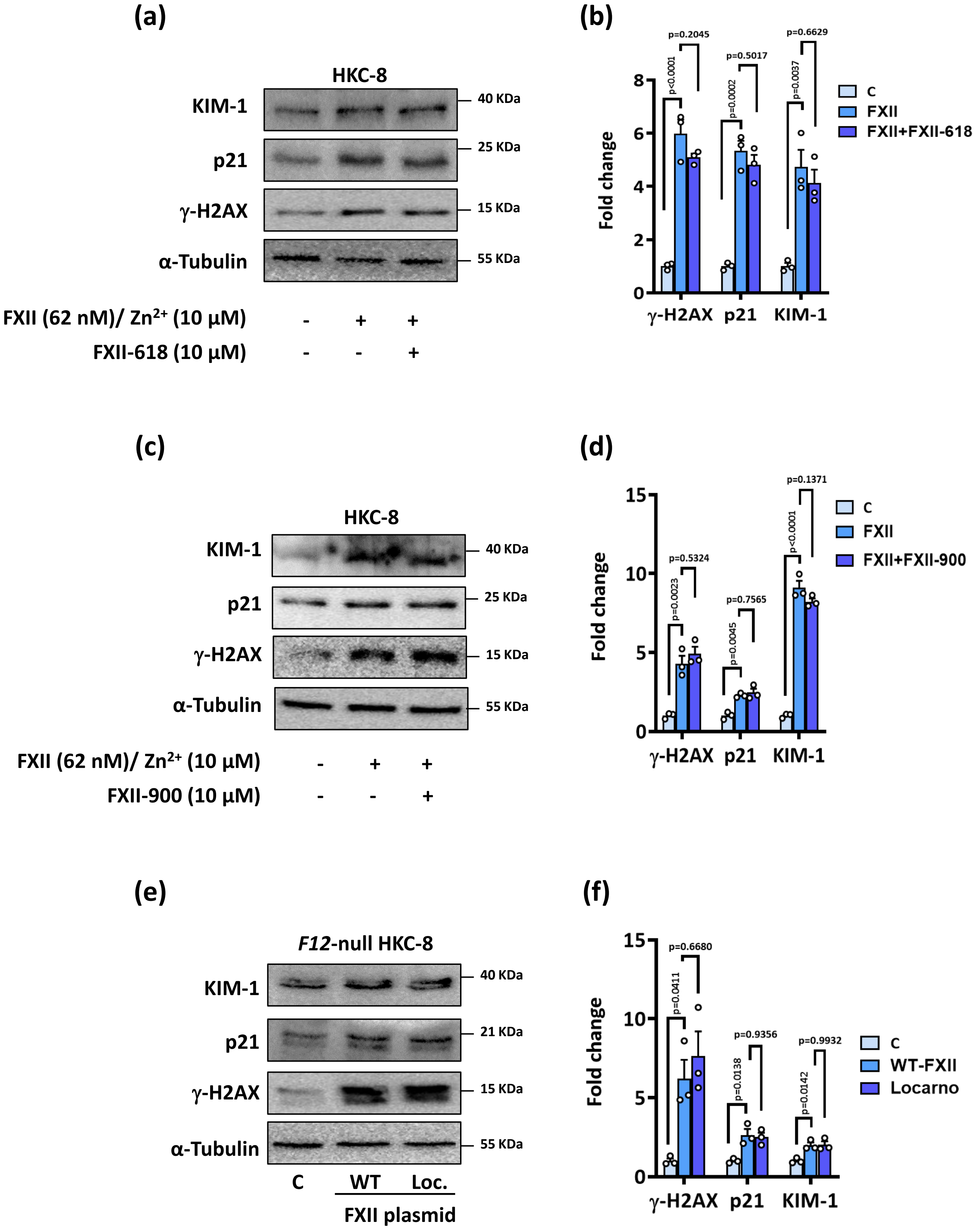
(a)
HKC-8
KIM-1
p21
γ-H2AX
α-Tubulin
40 KDa
25 KDa
15 KDa
55 KDa
FXII (62 nM)/ Zn2+ (10 µM) - + +
FXII-618 (10 µM) - - +
(b)
p=0.2045
p=0.5017
p=0.6629
p<0.0001
p=0.0002
p=0.0037
Fold change
γ-H2AX p21 KIM-1
C
FXII
FXII+FXII-618
(c)
HKC-8
KIM-1
p21
γ-H2AX
α-Tubulin
40 KDa
25 KDa
15 KDa
55 KDa
FXII (62 nM)/ Zn2+ (10 µM) - + +
FXII-900 (10 µM) - - +
(d)
p=0.1371
p=0.5324
p=0.7565
p=0.0023
p=0.0045
p<0.0001
Fold change
γ-H2AX p21 KIM-1
C
FXII
FXII+FXII-900
(e)
F12-null HKC-8
KIM-1
p21
γ-H2AX
α-Tubulin
40 KDa
21 KDa
15 KDa
55 KDa
C
WT
Loc.
FXII plasmid
(f)
p=0.6680
p=0.9356
p=0.9932
p=0.0411
p=0.0138
p=0.0142
Fold change
γ-H2AX p21 KIM-1
C
WT-FXII
Locarno

Fig. S14 FXII effects on tubular cells are independent on its enzymatic activity

a,b) Representative immunoblots (loading control: α-Tubulin, a) and dot plot summarizing results (b) for γ-H2AX, p21, and KIM-1 expression in HKC-8 cells exposed to purified human FXII (62 nM) in the presence of Zn^{2+} (10 µM) for 24 h with and without pretreatment with the active FXII cyclic peptide inhibitor; FXII-618 (10 µM) for 30 min. Dot plots reflecting mean±SEM of 3 independent experiments; one-way ANOVA with Tukeys's multiple comparison test.

c,d) Representative immunoblots (loading control: α-Tubulin, c) and dot plot summarizing results (d) for γ-H2AX, p21, and KIM-1 expression in HKC-8 cells exposed to purified human FXII (62 nM) in the presence of Zn^{2+} (10 µM) for 24 h with and without pretreatment with the active FXII cyclic peptide inhibitor; FXII-900 (10 µM) for 30 min. Dot plots reflecting mean±SEM of 3 independent experiments; one-way ANOVA with Tukeys's multiple comparison test.

e,f) Representative immunoblots (loading control: α-Tubulin, e) and dot plot summarizing results (f) for γ-H2AX, p21, and KIM-1 expression in *F12*-null HKC-8 cells transfected with wild type FXII (WT-FXII) or the mutant Locarno FXII lacking enzymatic activity (Loc. FXII) compared to empty-vector transfected cells. Dot plots reflecting mean±SEM of 3 independent experiments; one-way ANOVA with Tukeys's multiple comparison test.

Fig. S.15

(a)

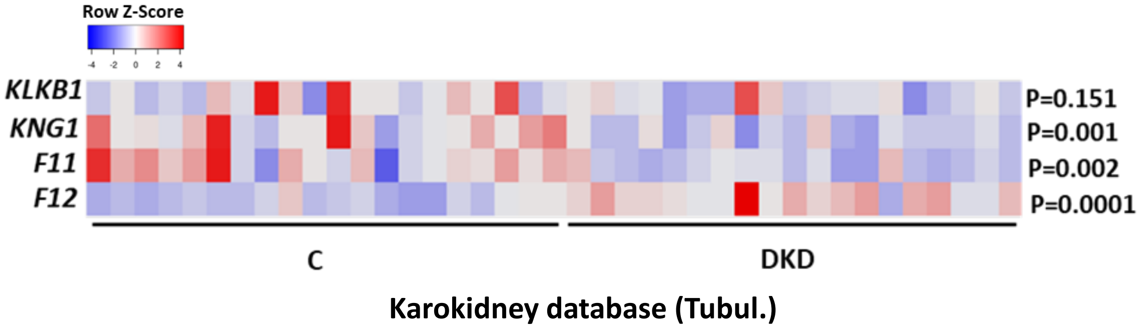
Row Z-Score
-4 -2 0 2 4
KLKB1
KNG1
F11
F12
P=0.151
P=0.001
P=0.002
P=0.0001
C
DKD
Karokidney database (Tubul.)

(b)

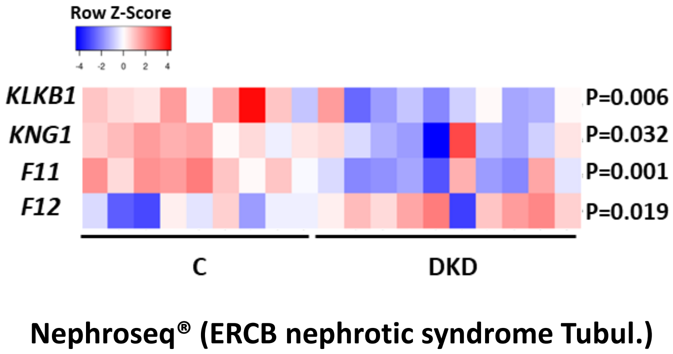
Row Z-Score
-4 -2 0 2 4
KLKB1
KNG1
F11
F12
P=0.006
P=0.032
P=0.001
P=0.019
C
DKD
Nephroseq® (ERCB nephrotic syndrome Tubul.)

(c)

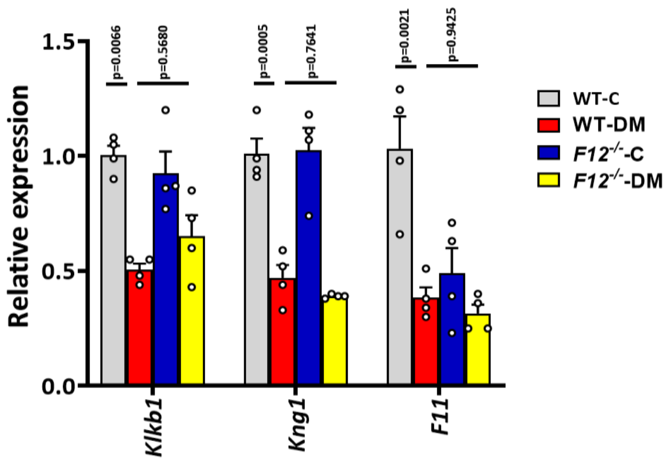
Relative expression
1.5
1.0
0.5
0.0
p=0.0066
p=0.5680
p=0.0005
p=0.7641
p=0.0021
p=0.9425
Klkb1
Kng1
F11
WT-C
WT-DM
F12-/--C
F12-/--DM

(d)

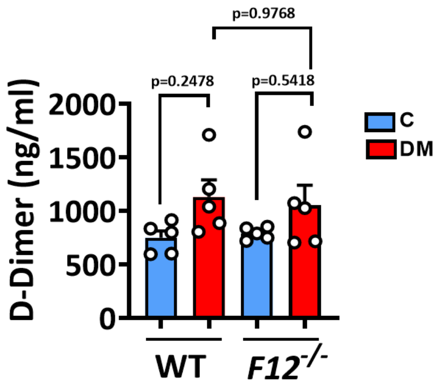
D-Dimer (ng/ml)
2000
1500
1000
500
0
p=0.9768
p=0.2478
p=0.5418
WT
F12-/-
C
DM

Fig. S15 FXII effects on tubular cells are not related to the protease procoagulant or proinflammatory functions

a,b) Heatmaps summarizing *KLKB1*, *KNG1*, *F11*, and *F12* expression in the tubulointerstitial compartment comparing diabetic kidney disease patients (DKD) to non-diabetic control healthy donors (C) in Karokidney database (a) and ERCB nephrotic syndrome of the Nephroseq® database (b). Two-tailed, unpaired student's t-test. Same datasets of *F12* and *KNG1* as in Fig. S11a,b were used to generate the heatmaps. Color intensity represents row Z-score.

c) Dot plots representing *Klkb1, Kng1 and F11* expression in normoglycemic controls (C) and hyperglycemic (DM) wild type (WT) and $F12^{-/-}$ mice (qPCR). Dot plots reflecting mean±SEM of 4 mice per group; two-tailed unpaired student's t-test.

d) dot plot summarizing plasma levels of D-Dimer in experimental groups (as described in c). Dot plot reflecting mean±SEM of 5 mice per group; two-way ANOVA with Tukeys's multiple comparison test.

Fig. S.16

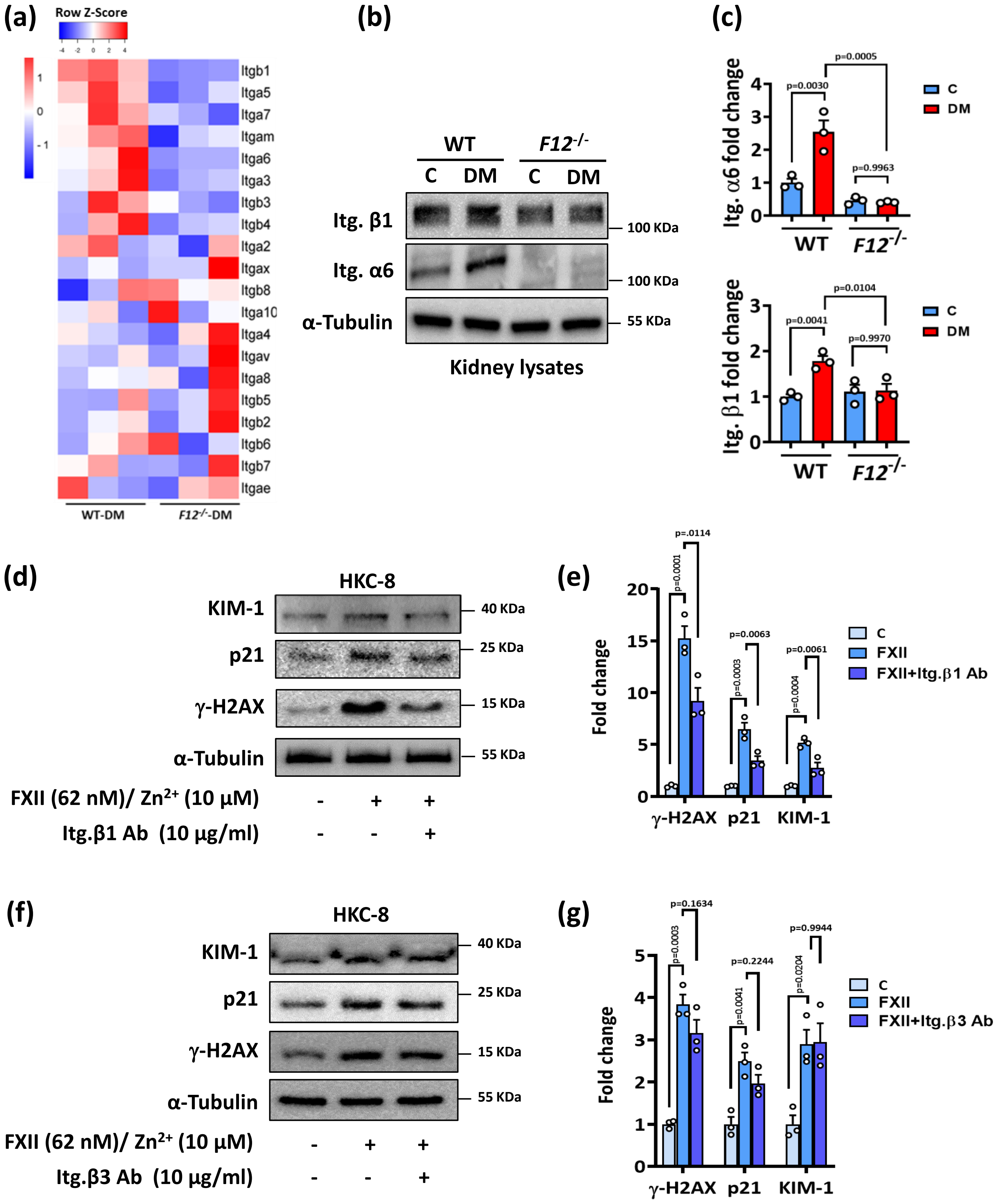

(a)
Row Z-Score
Itgb1
Itga5
Itga7
Itgam
Itga6
Itga3
Itgb3
Itgb4
Itga2
Itgax
Itgb8
Itga10
Itga4
Itgav
Itga8
Itgb5
Itgb2
Itgb6
Itgb7
Itgae
WT-DM
F12-/--DM
(b)
WT
F12-/-
C DM C DM
Itg. β1
Itg. α6
α-Tubulin
100 KDa
100 KDa
55 KDa
Kidney lysates
(c)
Itg. α6 fold change
p=0.0030
p=0.0005
p=0.9963
C
DM
WT
F12-/-
Itg. β1 fold change
p=0.0041
p=0.0104
p=0.9970
(d)
HKC-8
KIM-1
p21
γ-H2AX
α-Tubulin
40 KDa
25 KDa
15 KDa
55 KDa
FXII (62 nM)/ Zn2+ (10 μM) - + +
Itg.β1 Ab (10 μg/ml) - - +
(e)
Fold change
p=.0114
p=0.0001
p=0.0063
p=0.0003
p=0.0061
p=0.0004
C
FXII
FXII+Itg.β1 Ab
γ-H2AX p21 KIM-1
(f)
HKC-8
KIM-1
p21
γ-H2AX
α-Tubulin
40 KDa
25 KDa
15 KDa
55 KDa
FXII (62 nM)/ Zn2+ (10 μM) - + +
Itg.β3 Ab (10 μg/ml) - - +
(g)
Fold change
p=0.1634
p=0.0003
p=0.2244
p=0.0041
p=0.9944
p=0.0204
C
FXII
FXII+Itg.β3 Ab
γ-H2AX p21 KIM-1

Fig. S16 uPAR-integrin β1 interaction promotes FXII-dependent tubular injury

a) Heatmap of the RNA-seq data showing the expression of different integrins in WT-DM and *F12*$^{-/-}$-DM mice. Each column represents data from an individual mouse. Color intensity represents row Z-score.

b,c) Representative immunoblots (b, loading control: α-Tubulin) and dot plots summarizing results (c) for Itg. β1 and α6 expression in kidney lysates of normoglycemic controls (C) and hyperglycemic (DM) wild type (WT) and F12$^{-/-}$ mice. Dot plots reflecting mean±SEM of 3 mice per group; two-way ANOVA with Tukeys's multiple comparison test.

d,e) Representative immunoblots (loading control: α-Tubulin, d) and dot plot summarizing results (e) for γ-H2AX, p21, and KIM-1 expression in HKC-8 cells exposed to purified human FXII (62 nM) in the presence of Zn^{2+} (10 µM) for 24 h with and without pretreatment with integrin β1 blocking monoclonal antibody (10 µg/ml) for 30 min. Dot plots reflecting mean±SEM of 3 independent experiments; one-way ANOVA with Tukeys's multiple comparison test.

f,g) Representative immunoblots (loading control: α-Tubulin, f) and dot plot summarizing results (g) for γ-H2AX, p21, and KIM-1 expression in HKC-8 cells exposed to purified human FXII (62 nM) in the presence of Zn^{2+} (10 µM) for 24 h with and without pretreatment with integrin β3 blocking monoclonal antibody (10 µg/ml) for 30 min. Dot plots reflecting mean±SEM of 3 independent experiments; one-way ANOVA with Tukeys's multiple comparison test.

Fig. S.17

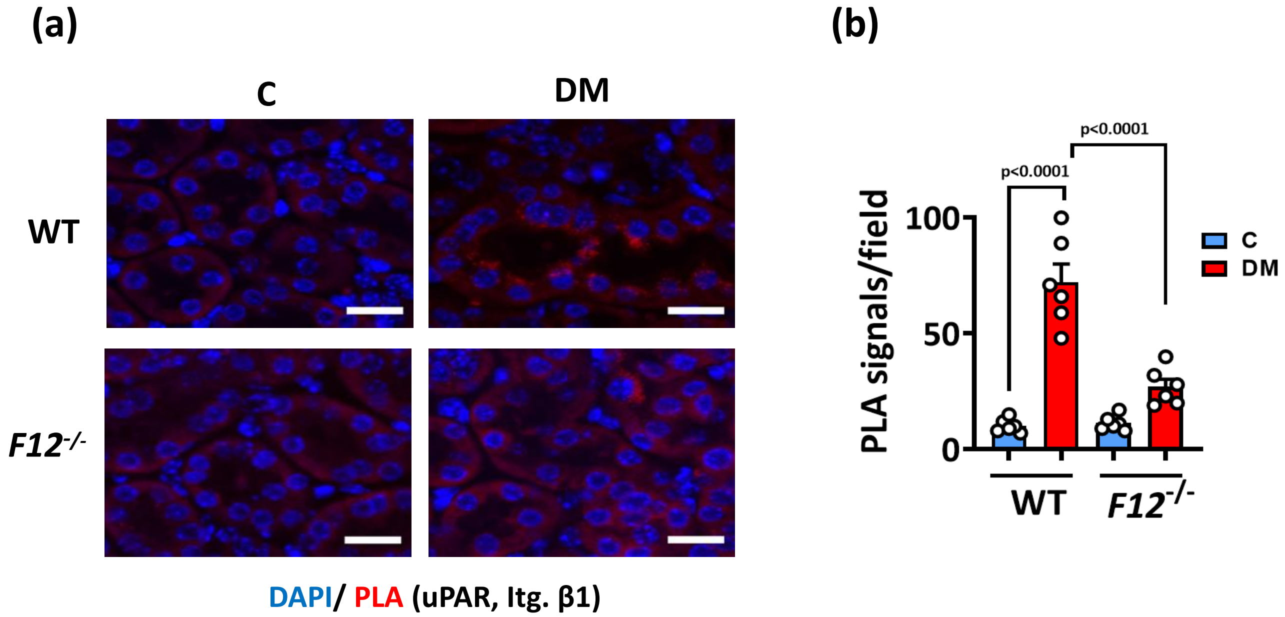

(a)
C
DM
WT
F12-/-
DAPI/ PLA (uPAR, Itg. β1)
(b)
p<0.0001
p<0.0001
PLA signals/field
100
50
0
C
DM
WT
F12-/-

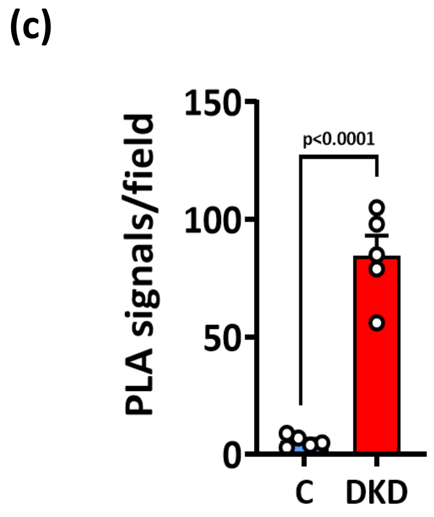

(c)
150
100
50
0
p<0.0001
PLA signals/field
C
DKD

Fig. S17 FXII promotes uPAR-integrin β1 interaction *in vivo*

a,b) Representative images of proximity ligation assay (PLA, d; uPAR and active integrin β1 interaction, red, d) and dot plot summarizing results (e) in normoglycemic controls (C) and hyperglycemic (DM) WT and $F12^{-/-}$ mice; DAPI nuclear counterstain, blue. Scale bars represent 20 µm. Dot plot reflecting mean±SEM of 6 mice per group with each dot representing the mean of PLA signals/field for one mouse; two-way ANOVA with Tukeys's multiple comparison test.

c) Dot plot summarizing quantification of proximity ligation assay (PLA, exemplary images Fig. 8h, uPAR and active integrin β1 interaction) in human kidney sections obtained from non-diabetic controls (C) or diabetic patients with DKD (DKD). Dot plot reflecting mean±SEM of 5 samples per group with each dot representing the mean of PLA signals/field for one sample; two-tailed unpaired student's t-test.

Fig. S.18

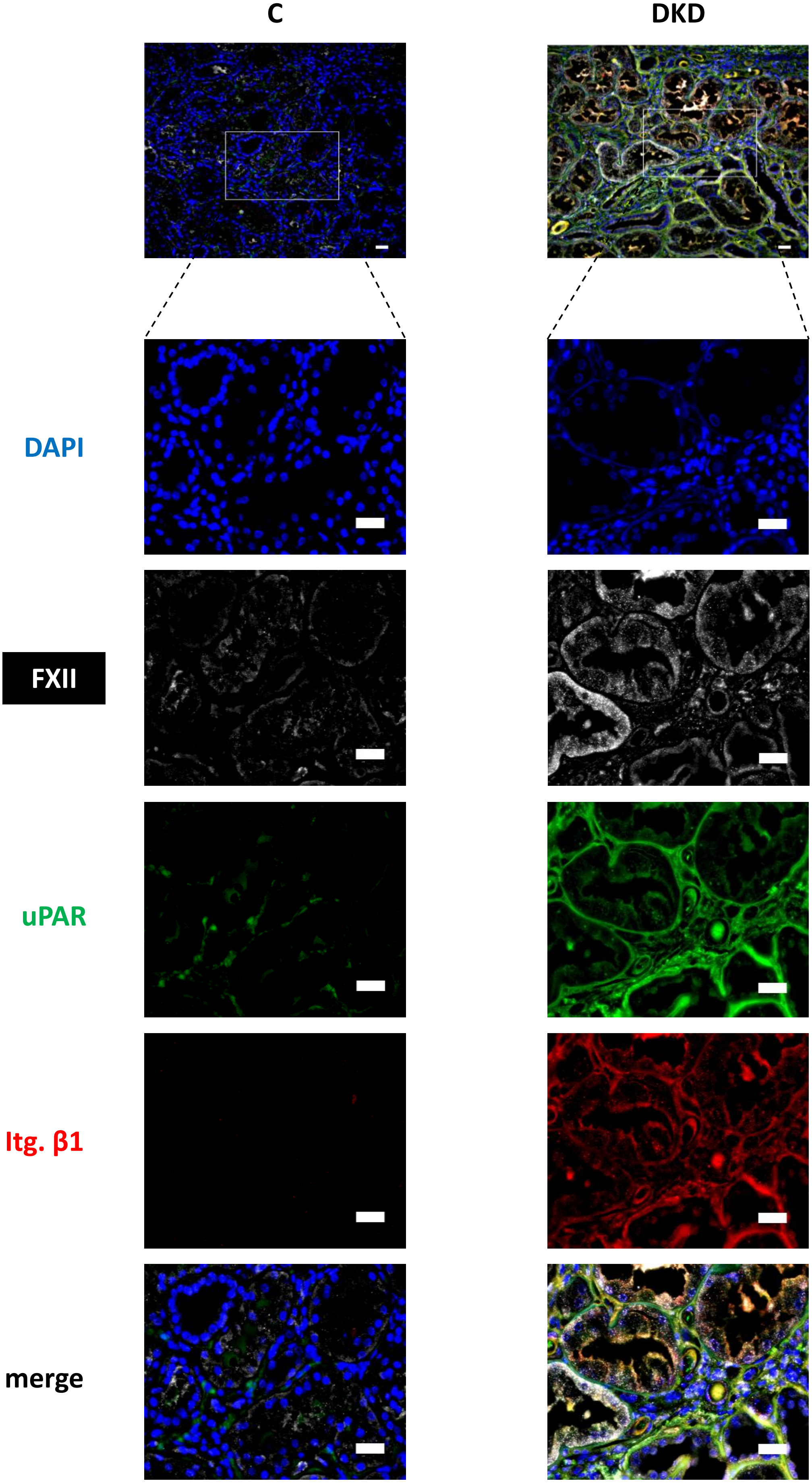

Fig. S18 FXII interacts with uPAR and integrin β1 in human DKD

Representative histological images of immunostaining of FXII (white), uPAR (green) and active integrin β1 in human kidney sections of nondiabetic controls (C) or diabetic patients with DKD (DKD); DAPI nuclear counterstain, (blue). Scale bars represent 20 µm. Representative images of 5 samples per group.

Fig. S.19

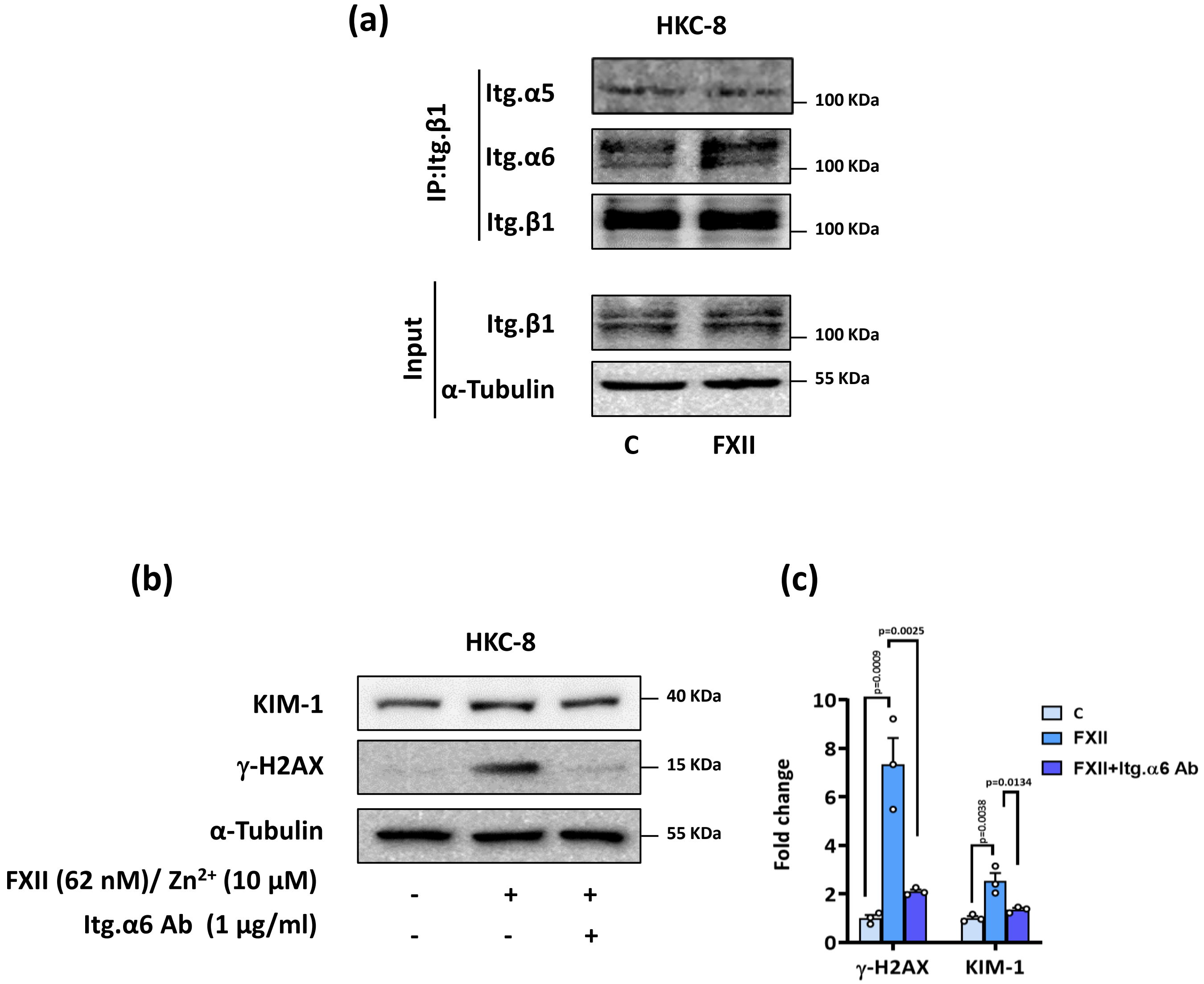
(a)
HKC-8
IP:Itg.β1
Itg.α5
Itg.α6
Itg.β1
100 KDa
100 KDa
100 KDa
Input
Itg.β1
α-Tubulin
100 KDa
55 KDa
C
FXII
(b)
HKC-8
KIM-1
γ-H2AX
α-Tubulin
40 KDa
15 KDa
55 KDa
FXII (62 nM)/ Zn2+ (10 μM)
Itg.α6 Ab (1 μg/ml)
(c)
p=0.0025
p=0.0009
p=0.0134
p=0.0038
Fold change
γ-H2AX
KIM-1
C
FXII
FXII+Itg.α6 Ab

Fig. S19 FXII promotes integrin α6β1 heterodimer formation in tubular cells

(a) Representative immunoblots for integrin α6 and integrin α5 from integrin β1 coimmunoprecipitation (IP, top) and immunoblots for integrin β1 and α-Tubulin from the input (input, bottom,) of HKC-8 cells exposed to purified human FXII (62nM) in the presence of Zn^{2+} (10 µM) for 24 h (FXII) compared to control non-treated cells (C). Input serves as a loading control. Immunoblots represent 3 independent experiments.

(b,c) Representative immunoblots (b, loading control: α-Tubulin) and dot plot summarizing results (c) for γ-H2AX and KIM-1 expression in HKC-8 cells exposed to purified human FXII (62nM) in the presence of Zn^{2+} (10 µM) for 24 h with and without pretreatment with a monoclonal integrin α6 function blocking antibody (1 µg/ml) for 30 min. Dot plots reflecting mean±SEM of 3 independent experiments; one-way ANOVA with Tukeys's multiple comparison test.

Fig. S.20

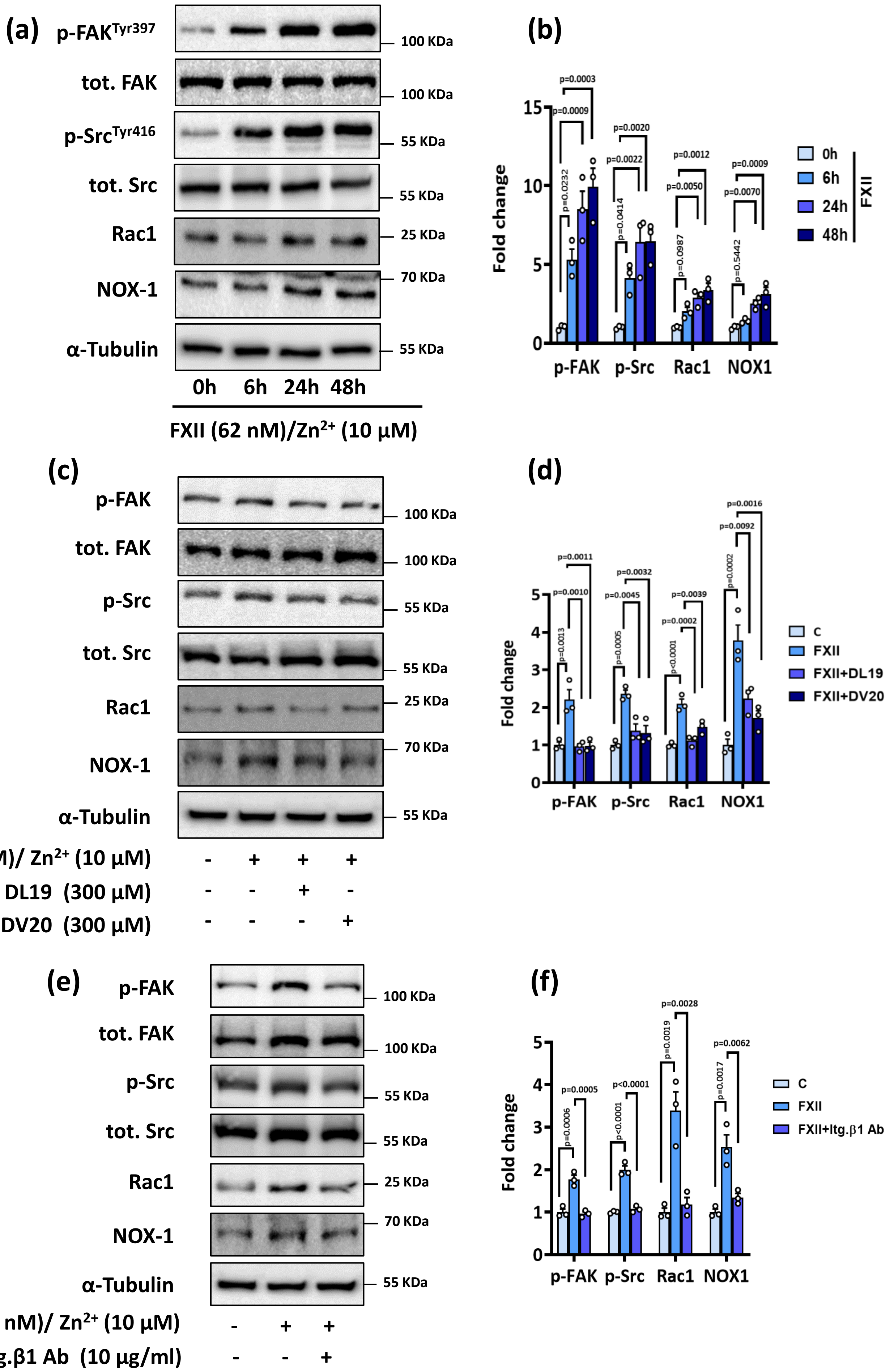

(a)
p-FAK Tyr397
tot. FAK
p-Src Tyr416
tot. Src
Rac1
NOX-1
α-Tubulin
100 KDa
100 KDa
55 KDa
55 KDa
25 KDa
70 KDa
55 KDa
0h 6h 24h 48h
FXII (62 nM)/Zn2+ (10 μM)
(b)
Fold change
p=0.0003
p=0.0009
p=0.0232
p=0.0020
p=0.0022
p=0.0414
p=0.0012
p=0.0050
p=0.0987
p=0.0009
p=0.0070
p=0.5442
p-FAK p-Src Rac1 NOX1
0h
6h
24h
48h
FXII
(c)
p-FAK
tot. FAK
p-Src
tot. Src
Rac1
NOX-1
α-Tubulin
100 KDa
100 KDa
55 KDa
55 KDa
25 KDa
70 KDa
55 KDa
FXII (62 nM)/ Zn2+ (10 μM) - + + +
DL19 (300 μM) - - + -
DV20 (300 μM) - - - +
(d)
Fold change
p=0.0011
p=0.0010
p=0.0013
p=0.0032
p=0.0045
p=0.0005
p=0.0039
p=0.0002
p<0.0001
p=0.0016
p=0.0092
p=0.0002
p-FAK p-Src Rac1 NOX1
C
FXII
FXII+DL19
FXII+DV20
(e)
p-FAK
tot. FAK
p-Src
tot. Src
Rac1
NOX-1
α-Tubulin
100 KDa
100 KDa
55 KDa
55 KDa
25 KDa
70 KDa
55 KDa
FXII (62 nM)/ Zn2+ (10 μM) - + +
Itg.β1 Ab (10 μg/ml) - - +
(f)
Fold change
p=0.0005
p=0.0006
p<0.0001
p<0.0001
p=0.0028
p=0.0019
p=0.0062
p=0.0017
p-FAK p-Src Rac1 NOX1
C
FXII
FXII+Itg.β1 Ab

Fig. S20 FXII promotes oxidative DNA damage in tubular cells through activation of focal adhesions

(a,b) Representative immunoblots (a, loading control: α-Tubulin) and dot plots summarizing results (b) for p-FAK, total FAK p-Src, total Src, Rac1 and NOX1 expression in HKC-8 cells exposed to purified human FXII (62nM) in the presence of Zn^{2+} (10 µM) for 6, 24, and 48 h. Dot plots reflecting mean±SEM of 3 independent experiments; one-way ANOVA with Tukeys's multiple comparison test.

(c,d) Representative immunoblots (c, loading control: α-Tubulin) and dot plots summarizing results (d) for p-FAK, total FAK p-Src, total Src, Rac1 and NOX1 expression in HKC-8 cells exposed to purified human FXII (62nM) in the presence of Zn^{2+} (10 µM) for 24 h with and without pretreatment with peptides DL19 and DV20 (300µM) for 1 h compared to control non-treated cells. Dot plots reflecting mean±SEM of 3 independent experiments; one-way ANOVA with Tukeys's multiple comparison test.

(e,f) Representative immunoblots (e, loading control: α-Tubulin) and dot plots summarizing results (f) for p-FAK, total FAK p-Src, total Src, Rac1 and NOX1 expression in HKC-8 cells exposed to purified human FXII (62nM) in the presence of Zn^{2+} (10 µM) for 24 h with and without pretreatment with a monoclonal integrin β1 function blocking antibody (10 µg/ml) for 30 min. compared to control non-treated cells. Dot plots reflecting mean±SEM of 3 independent experiments; one-way ANOVA with Tukeys's multiple comparison test.

Fig. S.21

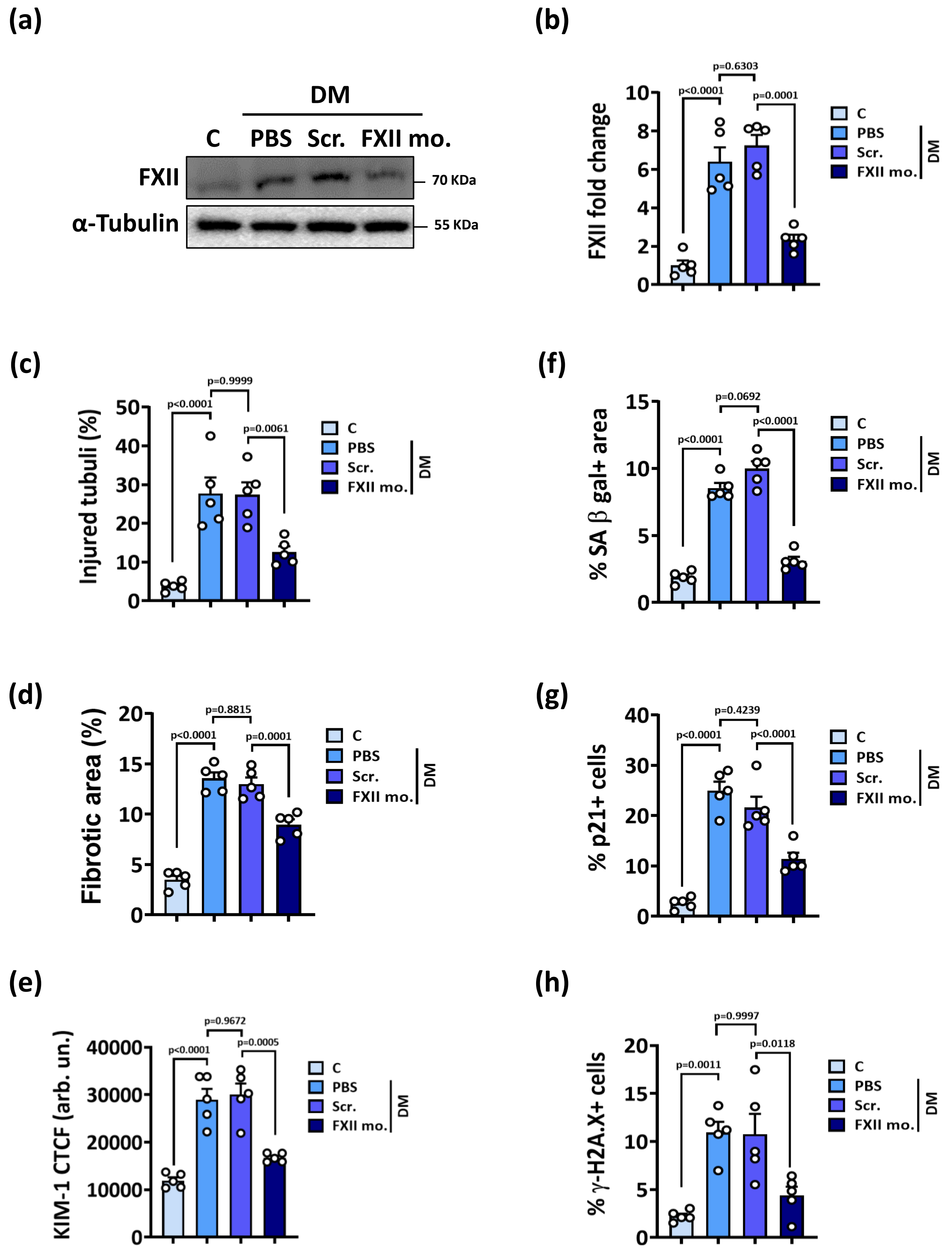
(a)
DM
C
PBS
Scr.
FXII mo.
FXII
70 KDa
α-Tubulin
55 KDa
(b)
FXII fold change
p<0.0001
p=0.6303
p=0.0001
(c)
Injured tubuli (%)
p<0.0001
p=0.9999
p=0.0061
(f)
% SA β gal+ area
p<0.0001
p=0.0692
p<0.0001
(d)
Fibrotic area (%)
p<0.0001
p=0.8815
p=0.0001
(g)
% p21+ cells
p<0.0001
p=0.4239
p<0.0001
(e)
KIM-1 CTCF (arb. un.)
p<0.0001
p=0.9672
p=0.0005
(h)
% γ-H2A.X+ cells
p=0.0011
p=0.9997
p=0.0118
C
PBS
Scr.
FXII mo.
DM

Fig. S21 Targeting FXII *in vivo* ameliorates established murine DKD

a,b) Representative immunoblots (a, loading control: α-Tubulin) and dot plot summarizing results (b) for FXII expression in kidney lysates of hyperglycemic WT mice (DM) treated with PBS, mismatch morpholino (Scr.), or FXII translational blocking morpholino (FXII mo.) compared to normoglycemic mice (C). Dot plot reflecting mean±SEM of 5 mice per group; one-way ANOVA with Tukeys's multiple comparison test.

c-h) Dot plots summarizing quantification of percentage of injured tubuli (c), fibrotic area (d), KIM-1 intensity (e), senescence associated β-galactosidase positive area (SA-β-gal, f), p21 positive cells (g), and phosphorylated H2A histone X (γ-H2AX, h) in experimental groups (as described in a). Dot plots reflecting mean±SEM of 5 mice per group; one-way ANOVA with Tukeys's multiple comparison test. CTCF: corrected total cell fluorescence. Arb. un.: arbitrary units

Fig. S.22

(a)

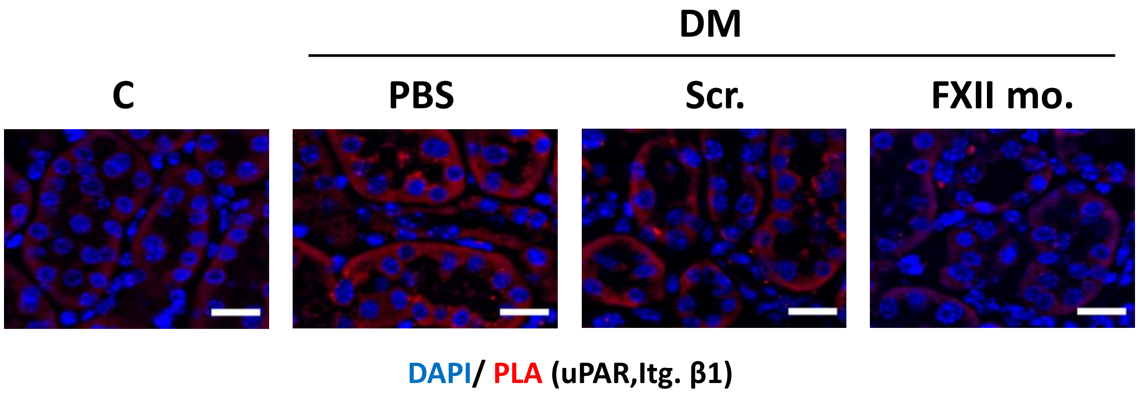

DM
C
PBS
Scr.
FXII mo.
DAPI/ PLA (uPAR,Itg. β1)

(b)

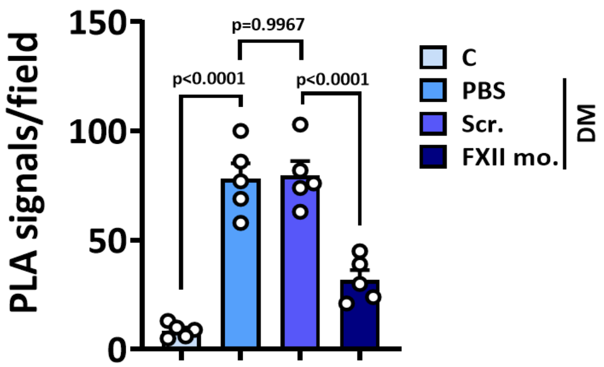

PLA signals/field
150
100
50
0
p=0.9967
p<0.0001
p<0.0001
C
PBS
Scr.
FXII mo.
DM

Fig. S22 Targeting FXII disrupts uPAR-integrin β1 interaction in murine DKD

a,b) Representative images of proximity ligation assay (PLA, a; uPAR and active integrin β1 interaction, red) and dot plot summarizing results (b) in hyperglycemic WT mice (DM) treated with PBS, mismatch morpholino (Scr.), or FXII translational blocking morpholino (FXII mo.) compared to normoglycemic mice (C); DAPI nuclear counterstain, blue. Scale bars represent 20 µm. Dot plot reflecting mean±SEM of 5 mice per group with each dot representing the mean of PLA signals/field for one mouse; one-way ANOVA with Tukeys's multiple comparison test.
