# Supplementary material for: Factor XII signaling via uPAR-integrin β1 axis promotes tubular senescence in diabetic kidney disease
Source: Nat Commun. 2024 Sep 11;15:7963. doi: 10.1038/s41467-024-52214-8 (PMC11390906; doi:10.1038/s41467-024-52214-8)
Supplement: Supplementary file 3 — Reporting Summary [file 41467_2024_52214_MOESM3_ESM.pdf]

nature portfolio

Corresponding author(s): Berend Isermann

Last updated by author(s): Aug 9, 2024

Reporting Summary

Nature Portfolio wishes to improve the reproducibility of the work that we publish. This form provides structure for consistency and transparency in reporting. For further information on Nature Portfolio policies, see our Editorial Policies and the Editorial Policy Checklist.

Statistics

For all statistical analyses, confirm that the following items are present in the figure legend, table legend, main text, or Methods section.

n/a	Confirmed	
☐	☒	The exact sample size (*n*) for each experimental group/condition, given as a discrete number and unit of measurement
☐	☒	A statement on whether measurements were taken from distinct samples or whether the same sample was measured repeatedly
☐	☒	The statistical test(s) used AND whether they are one- or two-sided<br>*Only common tests should be described solely by name; describe more complex techniques in the Methods section.*
☒	☐	A description of all covariates tested
☐	☒	A description of any assumptions or corrections, such as tests of normality and adjustment for multiple comparisons
☐	☒	A full description of the statistical parameters including central tendency (e.g. means) or other basic estimates (e.g. regression coefficient) AND variation (e.g. standard deviation) or associated estimates of uncertainty (e.g. confidence intervals)
☐	☒	For null hypothesis testing, the test statistic (e.g. *F*, *t*, *r*) with confidence intervals, effect sizes, degrees of freedom and *P* value noted<br>*Give P values as exact values whenever suitable.*
☒	☐	For Bayesian analysis, information on the choice of priors and Markov chain Monte Carlo settings
☒	☐	For hierarchical and complex designs, identification of the appropriate level for tests and full reporting of outcomes
☐	☒	Estimates of effect sizes (e.g. Cohen's *d*, Pearson's *r*), indicating how they were calculated

Our web collection on statistics for biologists contains articles on many of the points above.

Software and code

Policy information about availability of computer code

Data collection	Western Blotting: cseries capture software, Azure c400.<br>Microscopy: Leica Application Suite X (LAS X) software 8, Leica.<br>qRT-PCR: CFX Maestro Software 2.3.<br>Absorbance measurements: Gen5 software.<br>FACS.: Attune NxT flow cytometer.
Data analysis	Statistical analysis was performed using GraphPad Prism 9 software.<br>Images were analyzed using NIH Image J v1.48.<br>Differential gene expression analysis was conducted in Rstudio using DEbrowser by the DEseq2 R package.<br>Functional annotations and pathway analyses were perforemd using Gene Set Enrichment Analysis (GSEA 4.3.2) and ShinyGO 0.77.<br>FlowJo V.10 software was used to analyze FACS data.<br>Olink® NPX™ Signature software was used to anlyse Olink cytokine assay data.

For manuscripts utilizing custom algorithms or software that are central to the research but not yet described in published literature, software must be made available to editors and reviewers. We strongly encourage code deposition in a community repository (e.g. GitHub). See the Nature Portfolio guidelines for submitting code & software for further information.

Data

Policy information about availability of data

All manuscripts must include a data availability statement. This statement should provide the following information, where applicable:

- Accession codes, unique identifiers, or web links for publicly available datasets
- A description of any restrictions on data availability
- For clinical datasets or third party data, please ensure that the statement adheres to our policy

The data availability statement is given in the manuscript.

Research involving human participants, their data, or biological material

Policy information about studies with human participants or human data. See also policy information about sex, gender (identity/presentation), and sexual orientation and race, ethnicity and racism.

Reporting on sex and gender	Samples from both sexes were used in the analyses. The sex of the pariticipants was defined based on a self report. Distribution of sexes among the samples is provided in supplementary tables (S1-S3). All the collected samples were based on an informed consent. The inclusion criteria was type-2 diabetic patients with different grades of DKD and sex and age matched controls.
Reporting on race, ethnicity, or other socially relevant groupings	not applicable
Population characteristics	Population characteristics are mentioned within supplementary tables S1-S3 together with all the parameters tested.
Recruitment	Tissue samples were obtained from the National Center for Tumor Diseases (University of Heidelberg). Urine samples were obtained from the LIFE-biobank at the University Hospital of Leipzig which contained samples from the LIFE-ADULT study. Urine samples were obtained additonally from the HEIST-DiC study from the University hospital of Heidelberg. Urine samples were included in our study based on the inclusion criteria which was type-2 diabetic patietns with different grades of diabetic kidney disease and sex and age matched controls.
Ethics oversight	Human urine samples were obtained from the LIFE-ADULT cohort (Ethic vote no: 263-2009-14122009 and 201/17-ek, University of Leipzig) based on an informed consent.<br>Urine samples were obtained from HEIST-DiC study (Ethic vote no: S-383/2016, University of Heidelberg) based on an informed consent.

Note that full information on the approval of the study protocol must also be provided in the manuscript.

Field-specific reporting

Please select the one below that is the best fit for your research. If you are not sure, read the appropriate sections before making your selection.

[x] Life sciences [ ] Behavioural & social sciences [ ] Ecological, evolutionary & environmental sciences

For a reference copy of the document with all sections, see nature.com/documents/nr-reporting-summary-flat.pdf

Life sciences study design

All studies must disclose on these points even when the disclosure is negative.

Sample size	In all animal experiments, an estimate of the sample size was made based on effect size and power of the study using the software G-Power (v3.1.9.7).
Data exclusions	No data points were excluded.
Replication	The findings in human urine samples were replicated using two independent cohorts (LIFE-ADULT cohort and HEIST-DiC study), and using technical replicates. In case of in vitro experiments, three independent repeat experiments were performed. In case of in vivo experiments, a sample size was calculated (as descrived above) to ensure biological replicates.
Randomization	For in vivo experiments, mice were purchased or obtained from collaborators. Care was taken that each group contained age matched mice.<br>For human samples, samples were matched for key clinical features as shown in supplemetnary tables 1-3.<br>For in vitro experiments, cells were routinely passaged and randomly allocated for different treatment groups.
Blinding	The experimenter was blinded while performing the ex-vivo analysis of the mice data. However, blinding was not considered while performing in vivo interventions. This was done to ensure that the correct intervention was applied to each group as allocated. This was also important to ensure animal ethical guidelines and scoring based on the interventions. For experiments other than those involving mice work, the experimenter was blinded to group allocation during data collection and analysis.

Reporting for specific materials, systems and methods

We require information from authors about some types of materials, experimental systems and methods used in many studies. Here, indicate whether each material, system or method listed is relevant to your study. If you are not sure if a list item applies to your research, read the appropriate section before selecting a response.

Materials & experimental systems

n/a	Involved in the study	
☐	☒	Antibodies
☐	☒	Eukaryotic cell lines
☒	☐	Palaeontology and archaeology
☐	☒	Animals and other organisms
☒	☐	Clinical data
☒	☐	Dual use research of concern
☒	☐	Plants

Methods

n/a	Involved in the study	
☒	☐	ChIP-seq
☐	☒	Flow cytometry
☒	☐	MRI-based neuroimaging

Antibodies

Antibodies used	mouse anti-human FXII (clone C-8; sc-376770), mouse anti-Integrin β1 (clone 12G10; sc-59827), and mouse anti-Integrin β1 (clone P5D2; sc-59827), mouse anti-Kininogen HC (clone H-5; sc-166631), rat anti-Integrin a6 (clone GOH3; sc-19622), rabbit anti- Integrin a5 (clone H-104; sc-10729) (Santa Cruz Biotechnology, Germany); rabbit anti-human uPAR (clone D4Q5S; 12863), rabbit anti-human p21 (clone 12D1; 2947), rabbit anti--H2A.X (clone 20E3; 9718), rabbit anti-Ki-67 (clone D3B5; 9129), rabbit anti-Lamin B1 (clone E6M5T; 17416), rabbit anti-Bcl-2 (clone D17C4; 3498), rabbit anti-Bcl-xL (clone 54H6; 2764), rabbit anti-phospho-FAK (Tyr397; 3283), rabbit anti-FAK (3285), rabbit anti-phospho-Src (Tyr416; clone D49G4; 6943), rabbit anti-Src (2108); rabbit anti-Rac1/Cdc42 (4651); rabbit anti-α-Tubulin (clone 11H10; 2125), rabbit anti-β-Actin (clone 13E5; 4970); goat anti-rabbit IgG HRP-linked (7074), horse anti-mouse IgG HRP-linked (7076), goat anti-rat IgG HRP-linked (7077), mouse anti-rabbit IgG (conformation specific) HRP-linked (clone L27A9; 3678), rabbit anti-mouse IgG (light-chain specific) HRP-linked (clone D3V2A; 58802) (Cell Signaling Technology, Germany); rabbit anti-NOX1 (PA1739) (BOSTER, USA); rabbit anti-KIM-1 (ab47635) and rabbit anti-mouse p21 (clone EPR18021; ab188224), rat anti-F4/80 (clone BM8; ab16911) (Abcam, USA); rabbit anti-uPAR (orb13650) and rabbit anti-FXII (orb48369) (Biorbyt, UK); goat anti-mouse uPAR (AF534), mouse anti- 8-O-dG (clone 15A3; 4354-MC-050), VisUCyte HRP polymer mouse/rabbit IgG antibody (VC002-025) (R&D systems, USA); rabbit anti-WT-1 (clone CEH-23; MAB20854; Abnova, Taiwan); mouse anti- Integrin β3 (clone AP-5; EBW107; Kerafast, USA); mouse anti-Integrin β3 (clone B3A; MAB2023Z; Sigma-Aldrich, Germany); rat anti-mouse Integrin β1 (clone 9EG7; 553715; BD Biosciences, Germany); rat anti-mouse uPAR (clone 109801; MA5-23853), DyLight™ 649 donkey anti-rabbit IgG (4064; Biolegend, USA); Alexa Fluor™ 568 donkey anti-rabbit IgG (A10042), Alexa Fluor™ Plus 647 donkey anti-rabbit IgG (A32795), Alexa Fluor™ 488 donkey anti-mouse IgG (A-21202), Alexa Fluor™ 568 anti-mouse IgG (A10037), Alexa Fluor™ 488 Phalloidin (A12379) (Thermo Fischer Scientific, Germany).
Validation	The antibodies used within the study were validated by the company. Wherever possible, antibodies were internally validated using overexpression or knockout tissues or cell lines.

Eukaryotic cell lines

Policy information about cell lines and Sex and Gender in Research

Cell line source(s)	Immortalized human-derived renal proximal tubular cells (HKC-8) were provided by Prof. Dr. Peter Mertens, Magdeburg Germany.<br>Mouse primary proximal tubular epithelial cells were isolated from male C57BL/6 mice.
Authentication	Cell lines were validated for species using species specific primers. Cell type specific markers were tested using RT-PCR.
Mycoplasma contamination	Cell lines were routinely tested negative for mycoplasma.
Commonly misidentified lines (See ICLAC register)	No commonly misidentified cell lines were used.

Animals and other research organisms

Policy information about studies involving animals; ARRIVE guidelines recommended for reporting animal research, and Sex and Gender in Research

Laboratory animals	Strain: C57BL/6, C57BLKsJ-db/+ (db/m), C57BL/KSJ-db (db/db), and F12-/- on C57BL/6 background.<br>Age: starting 8 weeks of age at the beginning of the experiments.<br>Mice included in the study were matched for age and intervention group.<br>Housing conditions: Temperature 21 °C ± 2, RH 55 % ± 15, 10 air changes, 12 h/12 h day/night, Barrier housing in open cages for breeding and husbandry purposes, as well as experimental areas.

Wild animals	No wild animals were used.
Reporting on sex	Only male F12-/- mice were provided by Prof. Dr. Thomas Renné, and hence we used male mice for all other intervention groups. Furthermore, male mice were used to exclude any sex based effect, which was not an aim of this project.
Field-collected samples	No field collected samples were used.
Ethics oversight	All animal experiments were conducted following standards and procedures approved by the local animal care and use committee (TVV 70/21, Landesverwaltungsamt, Leipzig, Germany).

Note that full information on the approval of the study protocol must also be provided in the manuscript.

Plants

Seed stocks	*Report on the source of all seed stocks or other plant material used. If applicable, state the seed stock centre and catalogue number. If plant specimens were collected from the field, describe the collection location, date and sampling procedures.*
Novel plant genotypes	*Describe the methods by which all novel plant genotypes were produced. This includes those generated by transgenic approaches, gene editing, chemical/radiation-based mutagenesis and hybridization. For transgenic lines, describe the transformation method, the number of independent lines analyzed and the generation upon which experiments were performed. For gene-edited lines, describe the editor used, the endogenous sequence targeted for editing, the targeting guide RNA sequence (if applicable) and how the editor was applied.*
Authentication	*Describe any authentication procedures for each seed stock used or novel genotype generated. Describe any experiments used to assess the effect of a mutation and, where applicable, how potential secondary effects (e.g. second site T-DNA insertions, mosiacism, off-target gene editing) were examined.*

Flow Cytometry

Plots

Confirm that:

☒ The axis labels state the marker and fluorochrome used (e.g. CD4-FITC).

☒ The axis scales are clearly visible. Include numbers along axes only for bottom left plot of group (a 'group' is an analysis of identical markers).

☒ All plots are contour plots with outliers or pseudocolor plots.

☒ A numerical value for number of cells or percentage (with statistics) is provided.

Methodology

Sample preparation	At the end of the treatments, HKC-8 cells were washed from the culture medium with PBS and harvested then fixed in 3.7% paraformaldehyde for 15 min at room temperature. Cells were washed with PBS and pelleted down, then stained with rabbit anti-uPAR (1:200; Biorbyt) prepared in 1% BSA in PBS for 1 h at room temperature. Cells were then washed in PBS and stained with DyLight™ 649 donkey anti-rabbit IgG (1:500; Biolegend) for 1 h at room temperature. Cells were washed in PBS, pelleted down and resuspended in 1% BSA in PBS and the mean fluorescence intensity (MFI) of uPAR surface expression was acquired using flow cytometry.
Instrument	Attune NxT; Thermo Fischer Scientific
Software	FlowJo V.10 software (BD Biosciences)
Cell population abundance	Pure population of HKC-8 cells were only observed.
Gating strategy	Cell scatter properties were identified using FSC-A and SSC-A to identify cell populations. Singlet cells were distinguished from aggregates by combining FSC-A and FFSC-H. The surface expression of uPAR was detected using an RL1-A Dylight 649-A labeled antibody. The analysis of uPAR's presence and expression levels on the cell surface was performed through the assessment of histograms and Mean Fluorescence Intensity (MFI) measurements.

☒ Tick this box to confirm that a figure exemplifying the gating strategy is provided in the Supplementary Information.
